# Alternative mRNA polyadenylation regulates macrophage hyperactivation via the autophagy pathway

**DOI:** 10.1038/s41423-024-01237-8

**Published:** 2024-11-13

**Authors:** Yunzhu Chen, Baiwen Chen, Jingyu Li, Haixin Li, Gaoyang Wang, Xuemin Cai, Qianqian Zhang, Xiaoxu Liu, Chen Kan, Lei Wang, Zhengting Wang, Hua-Bing Li

**Affiliations:** 1 Shanghai Institute of Immunology, State Key Laboratory of Oncogenes and Related Genes, Shanghai Jiao Tong University School of Medicine, Shanghai, China; 2 Shanghai Jiao Tong University School of Medicine - Yale Institute for Immune Metabolism, Shanghai Jiao Tong University School of Medicine, Shanghai, China; 3 Tongji University Cancer Center, Shanghai Tenth People’s Hospital, School of Medicine, Tongji University, Shanghai, China; 4 Institute of Immunological Innovation & Translation, Chongqing Medical University, Chongqing, China; 5 School of Biological Science, The University of Manchester, Manchester, UK; 6 Department of Geriatrics, Medical Center on Aging of Shanghai Ruijin Hospital, Shanghai Jiao Tong University School of Medicine, Shanghai, China; 7 Department of Gastroenterology, Ruijin Hospital, Shanghai Jiao Tong University School of Medicine, Shanghai, China; 8 Medical Center on Aging, Center for Immune-Related Diseases at Shanghai Institute of Immunology, Ruijin Hospital, Shanghai Jiao Tong University School of Medicine, Shanghai, China; 9 Chongqing International Institute for Immunology, Chongqing, China

**Keywords:** alternative polyadenylation, Nudt21, macrophage, autophagy, inflammation, Inflammation, Autophagy

## Abstract

Macrophage hyperactivation is a hallmark of inflammatory diseases, yet the role of alternative polyadenylation (APA) of mRNAs in regulating innate immunity remains unclear. In this study, we focused on 3’UTR-APA and demonstrated that Nudt21, a crucial RNA-binding component of the 3’UTR-APA machinery, is significantly upregulated in various inflammatory conditions. By utilizing myeloid-specific Nudt21-deficient mice, we revealed a protective effect of Nudt21 depletion against colitis and severe hyperinflammation, primarily through diminished production of proinflammatory cytokines. Notably, Nudt21 regulates the mRNA stability of key autophagy-related genes, *Map1lc3b* and *Ulk2*, by mediating selective 3’UTR polyadenylation in activated macrophages. As a result, Nudt21-deficient macrophages display increased autophagic activity, which leads to reduced cytokine secretion. Our findings highlight an unexplored role of Nudt21-mediated 3’UTR-APA in modulating macrophage autophagy and offer new insights into the modulation of inflammation and disease progression.

## Introduction

The inflammatory response is a vital component of the body’s immune defense, facilitating pathogen elimination and tissue healing [1]. However, dysregulation of this response can lead to chronic inflammatory conditions such as inflammatory bowel disease (IBD), rheumatoid arthritis (RA), psoriasis, and systemic lupus erythematosus (SLE), which are collectively known as immune-mediated inflammatory diseases (IMIDs) [2]. These conditions are characterized by aberrant immune responses and persistent inflammation, potentially escalating to severe, life-threatening conditions such as hemophagocytic lymphohistiocytosis (HLH) [3, 4].

Macrophage overactivation is a pivotal feature of IMIDs and contributes significantly to disease pathogenesis [5, 6]. Dysfunctional macrophages under these conditions exhibit heightened inflammatory responses, leading to tissue damage, sustained inflammation, and exacerbation of disease severity [7, 8]. There is an ongoing need to study the complex regulatory mechanisms that govern macrophage activation, which are crucial for making timely adjustments to prevent excessive inflammatory responses.

At the molecular level, precise transcriptional regulation and alterations in RNA metabolism are closely associated with macrophage activation states and cytokine production [9–12]. RNA metabolism includes various processes involved in RNA synthesis, processing, and degradation [13, 14]. Alternative polyadenylation (APA) is a critical process in RNA metabolism that generates mRNA isoforms with varying 3’ untranslated region (3’UTR) lengths by selecting distinct polyadenylation sites (PASs) [15, 16]. APA, which is closely associated with RNA splicing, occurs in two primary types: 3’UTR-APA, which adjusts 3’UTR length without altering coding sequences, and intronic-APA, which can produce truncated proteins. Our study specifically focused on 3’UTR-APA. Studies have shown that 3’UTR shortening occurs during macrophage differentiation or in response to viral stimuli [17–19]. However, the specific roles of 3’UTR-APA regulators, particularly their individual contributions to macrophage activation, remain unclear.

To explore this, we focused on Nudt21, a key RNA-binding protein involved in 3’UTR PAS selection [20, 21]. Loss of Nudt21 may promote the use of a proximal PAS, resulting in mRNAs with shorter 3’UTRs [22–24]. Our previous CRISPR screening results suggest that Nudt21 may act as a positive regulator of macrophage inflammatory activation [25]. Given the rapid changes in macrophage transcriptomes in response to stimuli, ascertaining the role of the APA regulator Nudt21 in modulating macrophage activation during inflammation is crucial.

Here, we found that Nudt21 mRNA expression was increased among different IMIDs. Consequently, we generated mice with lineage-specific deletion of Nudt21 in macrophages (*Nudt21*^*fl/fl*^ LysM^Cre^, synonymous with Nudt21-cKO) and documented a reduction in inflammation in two murine disease models—colitis and HLH—characterized by decreased proinflammatory cytokine production. We then focused on the mechanism underlying this regulation by conducting high-depth RNA sequencing and percentage of distal polyA site usage index (PDUI) analysis on bone marrow-derived macrophages (BMDMs) derived from Nudt21-cKO and WT mice. Notably, Nudt21 ablation promoted constitutive autophagy activation by regulating the mRNA stability of two key autophagy modulators, Map1lc3b and Ulk2, via the 3’UTR length of the mRNA. Our study unexpectedly revealed that Nudt21-mediated modulation of autophagy-related genes finely regulates macrophage autophagy, impacting inflammatory responses and disease progression.

## Results

### Increased expression of *NUDT21* in inflammatory diseases

**Fig. 1:**
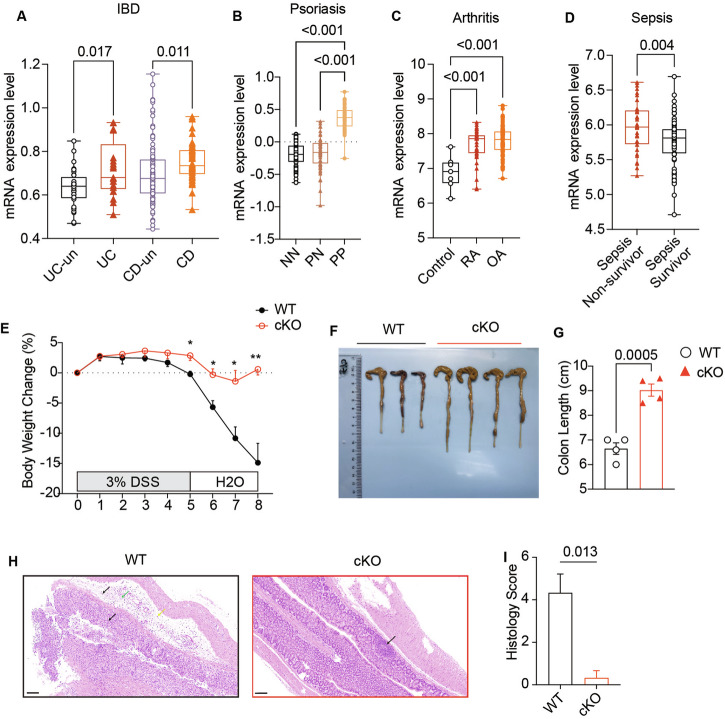
Increased *NUDT21* expression in inflammatory diseases and its protective role against colitis in Nudt21-deficient macrophages. mRNA expression levels of *NUDT21* in inflamed tissues from patients with inflammatory bowel disease (IBD) (GSE179285) (**A**), psoriasis (GSE13355) (**B**), rheumatoid arthritis (RA) and osteoarthritis (OA) (GSE236924) (**C**) and sepsis (GSE95233) (**D**). UC-un = uninflamed tissue from ulcerative colitis patients; UC = inflamed tissue from ulcerative colitis patients; CD-un = uninflamed tissue from Crohn’s disease patients; CD = inflamed tissue from Crohn’s disease patients; NN = normal skin from controls; PN = uninvolved skin from psoriatic patients; PP = involved skin from psoriatic patients; RA = rheumatoid arthritis; OA = osteoarthritis. Group sizes: (A) UC-un = 32; UC = 23, CD-un = 121, CD = 47, (B) NN = 64, PN = 58, PP = 58, **C** Control = 7, RA = 36, OA = 89, **D** Sepsis Nonsurvivor = 34, Sepsis Survivor = 68. Wild-type (WT) and Nudt21-cKO mice were treated with 3% dextran sulfate sodium (DSS) for 5 days followed by access to regular drinking water for 3 days. Body weight changes (**E**), representative images of the large intestine (**F**), colon length measurements (**G**), representative hematoxylin and eosin (H&E)-stained colon sections (**H**), and histological scoring (**I**) from both groups of mice were analyzed on day 8 after DSS treatment. Scale bars: 100 μm in **H**. The arrows indicate edema (yellow), lymphocytes (black), and neutrophils (green). The error bars represent the standard errors of the means (SEMs). *P* values were determined by unpaired Student’s *t*-test or one-way ANOVA

To explore the role of NUDT21 in hyperinflammatory conditions, we first analyzed the mRNA expression profiles of various immune-mediated inflammatory diseases (IMIDs) via the GEO database. Elevated levels of NUDT21 mRNA were observed in inflamed tissues from patients with inflammatory bowel disease (IBD) (Fig. 1A), psoriasis (Fig. 1B), rheumatoid arthritis (RA) (Fig. 1C), and sepsis (Fig. 1D, Fig. S1B). There was also a notable increase in expression in systemic lupus erythematosus (SLE) patients (Fig. S1C) and in peritoneal monocyte-derived macrophages from mice subjected to CAR-T-cell therapy-induced cytokine release syndrome (Fig. S1D). Additionally, Nudt21 was upregulated in bone marrow-derived macrophages (BMDMs) after IFNγ/LPS stimulation at both the mRNA and protein levels (Fig. S1E, F). These findings suggest that NUDT21-expressing macrophages may facilitate the progression of inflammation.

### Protective role of *Nudt21*-deficient macrophages against Colitis

To investigate the in vivo function of Nudt21 in macrophages, we generated myeloid-specific Nudt21-deficient mice (*Nudt21*^fl/fl^ LysM^Cre^, hereafter referred to as Nudt21-cKO) by crossing *Nudt21*-floxed mice (*Nudt21*^*fl/fl*^) with lysozyme M-Cre tool mice (LysM-Cre, referred to as WT) (Fig. S2A). RT‒qPCR and immunoblot analyses confirmed the efficient depletion of Nudt21 in bone marrow-derived macrophages (BMDMs) from Nudt21-cKO mice at both the mRNA and protein levels (Fig. S2B, C). Immune cell profiling revealed similar compositions of the spleen and bone marrow between Nudt21-cKO and WT mice (Fig. S2D, E).

To determine the functional impact of Nudt21 deficiency on macrophages during inflammatory disease progression, we subjected WT and Nudt21-cKO mice to a 3% dextran sulfate sodium (DSS) protocol [26–28] for five days to induce colitis. Compared with their WT counterparts, Nudt21-cKO mice presented significantly less body weight loss, reduced colon shortening, and milder colon inflammation (Fig. 1E–I) while maintaining comparable myeloid cell counts in both groups (Fig. S2F). These findings suggest that Nudt21 deficiency confers a protective effect against colitis in mice, primarily through alterations in macrophage function. The reduction in colitis severity in Nudt21-cKO mice underscores the critical role of Nudt21 in modulating macrophage-mediated inflammatory responses.

### Nudt21 loss in macrophages alleviates excessive inflammation

**Fig. 2:**
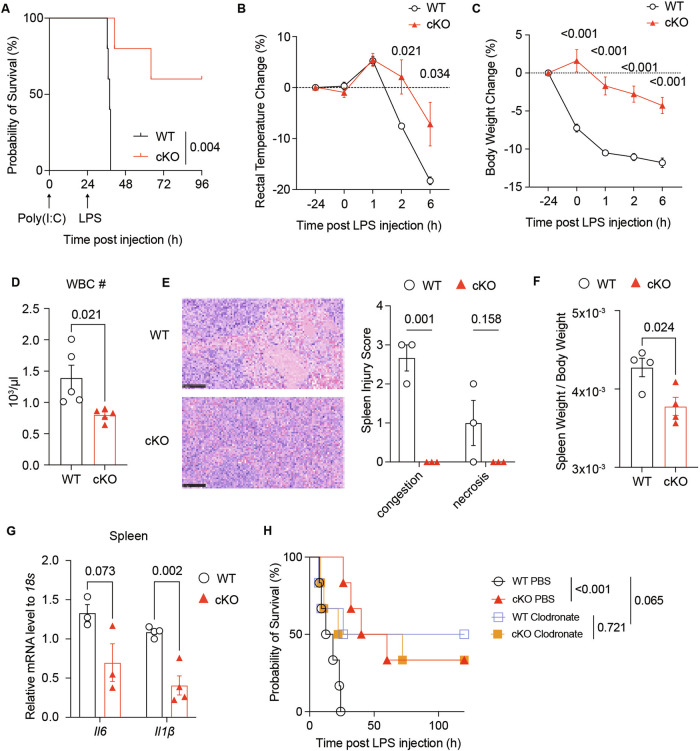
Loss of Nudt21 in Macrophages Alleviates Excessive Inflammation in an HLH Model. **A** Survival curves of WT and Nudt21-cKO mice in the hemophagocytic lymphohistiocytosis (HLH) model, with polyI:C (10 mg/kg) followed by lipopolysaccharide (LPS) (5 mg/kg) injections (*n* = 5 per group). Survival curves were analyzed via the log-rank (Mantel‒Cox) test. Analyses conducted 6 hours post-LPS injection included rectal temperature changes (**B**), body weight changes (**C**), white blood cell counts in peripheral blood (**D**), representative H&E-stained spleen sections and spleen injury scores (**E**) (*n* = 3 per group), the ratio of spleen weight to body weight (**F**), and the mRNA expression levels of Il6 and Il1β in spleen tissues (**G**). *P* values were determined by unpaired Student’s *t* test. (**H**) Survival curves of WT and Nudt21-cKO mice following the indicated treatments (*n* = 6 per group). Survival curves were analyzed via the log-rank (Mantel‒Cox) test. The error bars represent the standard errors of the means (SEMs)

To investigate the role of macrophage-specific Nudt21 deficiency in acute hyperinflammation, we subjected mice to a hemophagocytic lymphohistiocytosis (HLH) animal model induced by intraperitoneal injections of polyI:C for 24 hours followed by LPS [29–31]. Compared with their WT counterparts, Nudt21-cKO mice presented significantly improved outcomes, as evidenced by increased survival rates (Fig. 2A) and less pronounced decreases in rectal temperature (Fig. 2B) and body weight (Fig. 2C) after HLH model induction. Additionally, the HLH Nudt21-cKO mice presented lower peripheral white blood cell counts (Fig. 2D), lower spleen injury scores (Fig. 2E), decreased splenic cellularity (Fig. S3A), less splenomegaly (Fig. 2F), and higher peripheral platelet counts (Fig. S3B). Moreover, these mice presented decreased mRNA expression of the proinflammatory cytokines IL6 and IL1β in both spleen and liver tissues, along with reduced plasma levels of IL1β (Fig. 2G, Fig. S3C, D).

To assess whether the enhanced survival of Nudt21-cKO mice was attributable primarily to macrophage effects, we depleted macrophages via clodronate liposomes [32–35]. This intervention reduced mortality in WT mice under the HLH model to a rate comparable with that in Nudt21-cKO mice, highlighting that the absence of Nudt21 in macrophages plays a key role in mitigating acute inflammation (Fig. 2H). Collectively, these findings demonstrate that Nudt21 deficiency in macrophages significantly ameliorates inflammation progression.

### Reduced proinflammatory traits in Nudt21-deficient macrophages

To further explore the characteristics of macrophages lacking Nudt21 during acute inflammatory disease progression, we analyzed myeloid cell populations in the spleen in the HLH model. Our findings revealed increases in both the absolute number and percentage of splenic macrophages in Nudt21-cKO mice compared with WT mice (Fig. S3F, G), suggesting increased cell viability. Additionally, a decreased percentage of Annexin V + 7-AAD^+^ cells was observed in splenic macrophages from Nudt21-cKO mice (Fig. S3H, I), indicating reduced macrophage death.

**Fig. 3:**
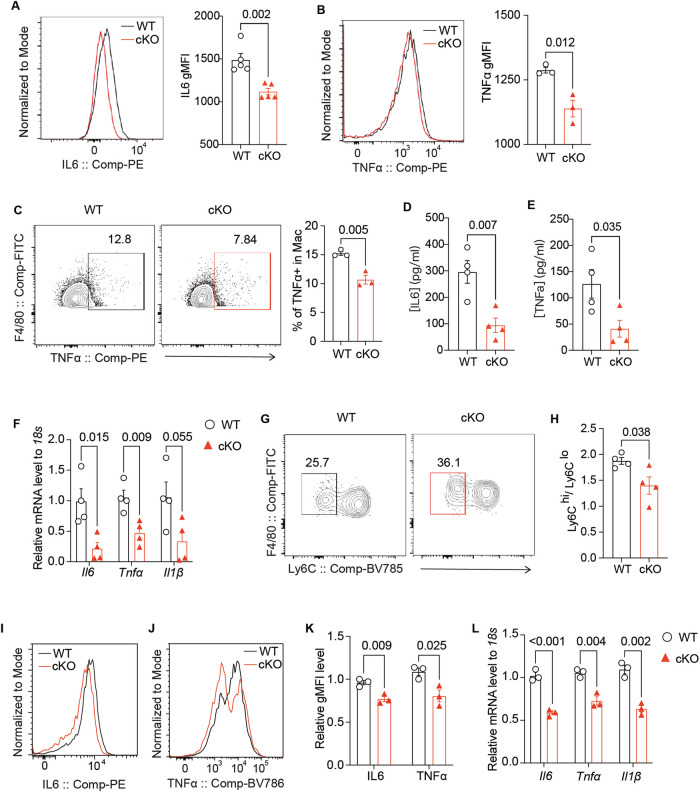
Reduced proinflammatory traits in Nudt21-deficient monocyte-derived macrophages. **A** Histogram illustrating the geometric mean fluorescence intensity (gMFI) of IL6 and quantification of the gMFI in splenic macrophages from WT and Nudt21-cKO mice in the HLH model (6 h, *n* = 5 per group). **B** Histogram displaying the gMFI of TNFα and quantification of the gMFI in splenic macrophages from WT and Nudt21-cKO mice in the HLH model (6 h, n = 3 per group). **C** Percentages of TNFα-positive macrophages in the spleens of WT and Nudt21-cKO mice in the HLH model (6 h, *n* = 3 per group). ELISA results showing IL6 (**D**) and TNFα (**E**) levels in the supernatants of splenic macrophages from HLH-WT and HLH-cKO mice 6 hours after LPS injection. **F** RT‒qPCR analysis of *Il6*, *Tnfα*, and *Il1β* in bone marrow monocytes from WT and Nudt21-cKO mice in the HLH model (6 h, *n* = 4 per group). Percentages of Ly6C^lo^ cells among monocyte-derived macrophages (**G**) and the ratio of Ly6C^hi^ to Ly6C^lo^ monocyte-derived macrophages (**H**) in the peritoneal cavities of WT and Nudt21-cKO mice in the HLH model (2 h, *n* = 4 per group). Histograms showing the gMFI of IL6 (**I**) and TNFα (**J**), along with the quantification (**K**) of bone marrow-derived macrophages (BMDMs) from WT and Nudt21-cKO mice primed with 50 ng/ml IFNγ overnight and 50 ng/ml LPS for 8 hours (*n* = 3 per group). **L** RT‒qPCR analysis of *Il6*, *Tnfα*, and *Il1β* in BMDMs from WT and Nudt21-cKO mice primed with 50 ng/ml IFNγ overnight and 50 ng/ml LPS for 8 hours (*n* = 3 per group). The error bars represent the standard errors of the means (SEMs). P values were determined by unpaired Student’s *t*-test

In terms of proinflammatory cytokines, splenic macrophages from Nudt21-cKO mice presented significantly lower levels of IL6 and TNFα (Fig. 3A, B), as did a reduced percentage of TNFα-positive macrophages (Fig. 3C). Consistently, in the supernatant of the splenic macrophages sorted from the HLH model mice, the cKO group presented lower IL6 and TNFα levels than the WT group did (Fig. 3D, E). Furthermore, these mice presented decreased mRNA levels of *Il6*, *Tnfα*, and *Il1β* (Fig. 3F) and a reduction in the IL12β geometric mean fluorescence intensity (gMFI) in both bone marrow monocytes (BMMs) and peritoneal monocyte-derived macrophages (PMMs) (Fig. S4A, B). Additionally, in the peritoneal cavity of HLH Nudt21-cKO mice, there was a decrease in the proportion of Ly6C^hi^ to Ly6C^low^ macrophages (Fig. 3G, H). Similar results were observed in the DSS model, where compared with WT intestinal macrophages, Nudt21-cKO intestinal macrophages exhibited reduced production of TNFα and IL6 (Fig. S4C-F).

On the basis of these in vivo findings, we stimulated BMDMs with IFNγ/LPS and assessed cytokine levels and cell viability. Flow cytometry analysis revealed reduced expression levels of IL6 and TNFα in Nudt21-cKO BMDMs (Fig. 3I–K). RT‒qPCR analysis confirmed that the mRNA levels of *Il6*, *Tnfα*, and *Il1β* were lower in Nudt21-cKO BMDMs than in their WT counterparts (Fig. 3L). Additionally, the viability of Nudt21-cKO BMDMs was greater than that of WT BMDMs (Fig. S3J), which is consistent with the in vivo disease model results.

These findings underscore the critical role of Nudt21 in modulating macrophage proinflammatory activation, with Nudt21 deficiency leading to significant reductions in inflammatory responses and enhanced macrophage viability across multiple experimental models.

### Enhanced autophagy in Nudt21-ablated macrophages in response to inflammatory activation

**Fig. 4:**
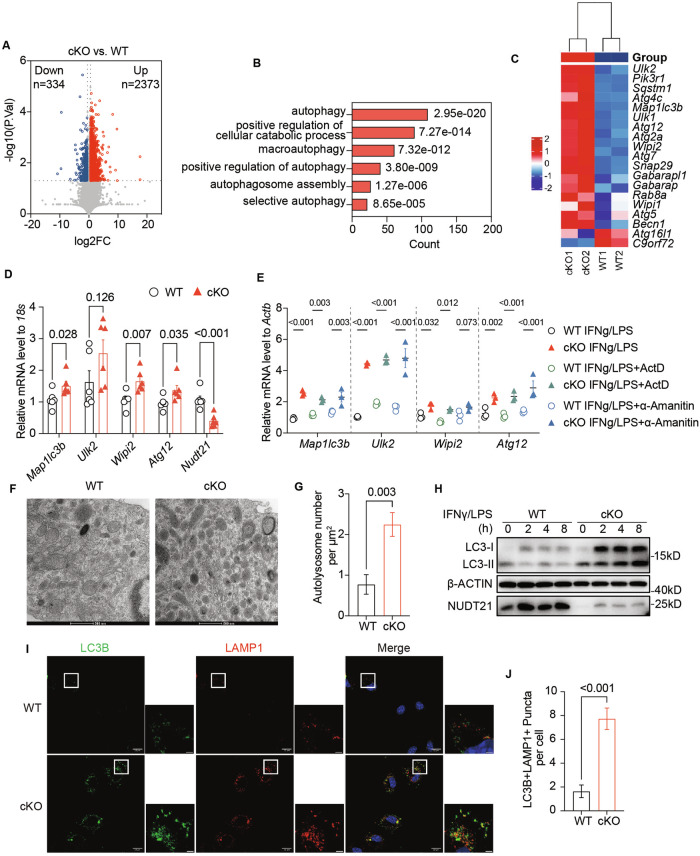
Enhanced Autophagy in Nudt21-Ablated Macrophages Following Inflammatory Activation. **A** Volcano plot showing DEGs in Nudt21-cKO BMDMs compared with WT BMDMs after treatment with IFNγ (50 ng/ml) overnight, followed by LPS (50 ng/ml) stimulation for 4 hours. Genes with a fold change greater than 1.5 and P < 0.05 are highlighted; upregulated genes are shown in red, and downregulated genes are shown in blue. **B** Gene Ontology (GO) enrichment analysis of transcripts upregulated in Nudt21-cKO BMDMs relative to WT controls after treatment with IFNγ (50 ng/ml) overnight and LPS (50 ng/ml) for 4 hours. **C** Heatmap displaying the expression profiles of genes associated with autophagy pathways in WT and Nudt21-cKO BMDMs following treatment with IFNγ (50 ng/ml) overnight and LPS (50 ng/ml) for 4 hours. **D** RT‒qPCR analysis of autophagy-related gene expression in bone marrow monocytes from WT and Nudt21-cKO mice in the HLH model (6 h, n = 6 per group). **E** RT‒qPCR analysis of the mRNA levels of autophagy-related genes in BMDMs from WT and Nudt21-cKO mice following treatment with IFNγ (50 ng/mL) overnight and LPS (50 ng/mL) for 4 hours, with or without 5 μM actinomycin D (ActD) or 5 μg/mL α-amanitin. Representative transmission electron microscopy (TEM) images (**F**) and quantification of autolysosomes (**G**) in BMDMs primed with 50 ng/ml IFNγ overnight and 50 ng/ml LPS for 4 hours. Scale bar: 500 nm. Images represent macrophages pooled from 2–3 mice per genotype and condition, with at least 6 images analyzed per genotype. **H** Immunoblot analysis showing the LC3 protein levels in WT and Nudt21-cKO BMDMs following overnight stimulation with IFNγ (50 ng/mL) and LPS (50 ng/mL) for the indicated durations. **I** Fluorescence microscopy images of LC3B and LAMP1 staining, along with DAPI counterstaining, in WT and Nudt21-cKO BMDMs primed with 50 ng/ml IFNγ overnight and 50 ng/ml LPS for 2 hours. Scale bar in the zoomed image: 2 µm. Captions represent at least two independent experiments. **J** Quantification of colocalized puncta of LC3 and LAMP1 across 50 cells per group. The error bars represent the standard errors of the means (SEMs). *P* values were determined by unpaired Student’s *t*-test

To investigate the molecular mechanism underlying the anti-inflammatory characteristics of Nudt21-deficient macrophages, we conducted high-throughput transcriptome sequencing (RNA-seq) on BMDMs from WT and Nudt21-cKO mice, both with and without IFNγ/LPS stimulation (RNA-seq, >3.0 × 10^8^ reads, 40 G raw data/sample). This analysis identified 2,707 differentially expressed genes, with 88% (2,373 genes) upregulated and 12% (334 genes) downregulated (Fig. 4A, Fig. S5A, B). Gene Ontology (GO) and Kyoto Encyclopedia of Genes and Genomes (KEGG) pathway analyses revealed that autophagy-related pathways were the most enriched (Fig. 4B, Fig. S5C, D). Compared with those from WT mice, BMMs from HLH Nudt21-cKO mice presented increased expression of key autophagy regulators, such as *Map1lc3b*, *Ulk2*, *Wipi2*, and *Atg12* (Fig. 4D). To explore whether Nudt21 regulates autophagy-related gene expression at the posttranscriptional level, we conducted RT‒qPCR analyses in WT and cKO BMDMs with or without the transcriptional inhibitors actinomycin D (ActD) and α-amanitin. Actinomycin D broadly inhibits transcription by preventing RNA polymerase movement [36, 37], whereas α-amanitin specifically targets RNA polymerase II, inhibiting mRNA synthesis [38]. Notably, even in the presence of these inhibitors, the mRNA levels of Map1lc3b, Ulk2, Wipi2, and Atg12 remained elevated in the cKO group (Fig. 4E). These results strongly suggest that Nudt21 plays a critical role in modulating the expression of these autophagy-related genes via posttranscriptional mechanisms.

Autophagy plays a crucial role in cellular homeostasis by orchestrating the degradation and recycling of cellular components [39, 40]. Dysfunctional autophagy in macrophages can disrupt the regulation of inflammatory cytokine production, exacerbating inflammatory diseases such as sepsis, inflammatory bowel disease, and atherosclerosis [41–43]. In our study, we assessed autophagic activity in Nudt21-cKO BMDMs upon IFNγ/LPS activation via a monodansylcadaverine (MDC) assay, which revealed an increase in the number of autophagic vacuoles (Fig. S5E). Transmission electron microscopy (TEM) analysis confirmed a significant increase in the number of autolysosomes in Nudt21-cKO BMDMs compared with WT controls (Fig. 4F, G). Additionally, immunoblotting revealed an elevated LC3II/LC3-I ratio in Nudt21-cKO BMDMs (Fig. 4H), indicating enhanced conversion of cellular LC3-I to the lipidated LC3-II form. Immunofluorescence staining further revealed pronounced colocalization of LC3 puncta with lysosomes in Nudt21-cKO BMDMs, unlike in the WT group (Fig. 4I, J).

These findings collectively indicate significantly enhanced autophagic activity in Nudt21-deficient macrophages, highlighting the vital role of Nudt21 in regulating autophagy during macrophage activation.

### Nudt21 modulates PolyA site selection and represses the mRNA stability of key autophagy genes

**Fig. 5:**
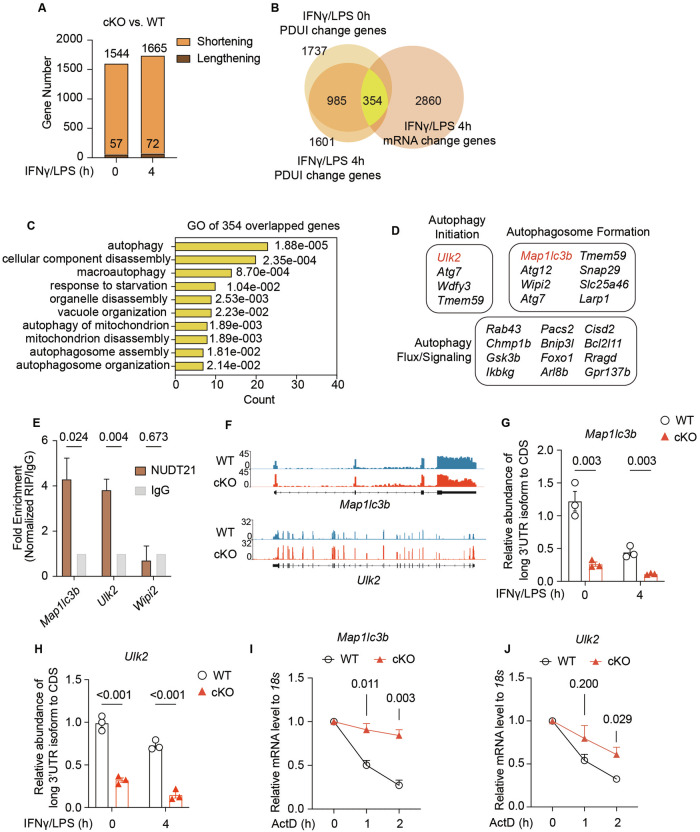
Nudt21 Directs PolyA Site Selection and Represses the mRNA Stability of Autophagy-Related Genes. **A** Histogram summarizing the number of genes associated with changes in the percent distal polyA site usage index (PDUI) in WT and Nudt21-cKO BMDMs primed with 50 ng/ml IFNγ overnight and 50 ng/ml LPS for the indicated durations. **B** Venn diagram showing the overlap of genes with changes in both 3’UTR length and mRNA expression levels in Nudt21-cKO BMDMs primed with 50 ng/ml IFNγ overnight and 50 ng/ml LPS for 4 hours. **C** Histogram of Gene Ontology (GO) enrichment analysis identifying autophagy-related pathways among the 354 genes with changes in PDUI and mRNA levels in Nudt21-cKO BMDMs compared with WT controls. **D** List of autophagy-related genes from the overlapping set shown in **B**. **E** RNA immunoprecipitation (RIP)-qPCR analysis showing the binding of Nudt21 to the mRNAs of Map1lc3b and Ulk2 in WT BMDMs primed with 50 ng/ml IFNγ overnight and 50 ng/ml LPS for 4 hours. **F** RNA-seq reads across the *Map1lc3b* and *Ulk2* loci in WT (blue) and Nudt21-cKO (red) BMDMs primed with 50 ng/ml IFNγ overnight and 50 ng/ml LPS for 4 hours. **G**, **H** RT‒qPCR analysis of distal polyA site usage in the 3’UTRs of *Map1lc3b* (G) and *Ulk2* (H) in WT and Nudt21-cKO BMDMs primed with 50 ng/ml IFNγ overnight and 50 ng/ml LPS for the indicated durations. **I**, **J** RT‒qPCR analysis of *Map1lc3b* (**J**) and *Ulk2* (**K**) mRNA decay in WT and Nudt21-cKO BMDMs at multiple time points (1 hour and 2 hours) after 5 μM actinomycin D (ActD) treatment (*n* = 3 per group). The error bars represent the standard errors of the means (SEMs). *P* values were determined by unpaired Student’s *t*-test

Nudt21 specifically recognizes UGUA motifs to facilitate the use of distal polyadenylation sites, and its absence leads to 3’ UTR shortening in mRNAs [21, 44, 45]. To investigate the effect of Nudt21 deletion on the 3’UTR length of mRNAs in activated macrophages, we utilized the DAPARS algorithm [46] to analyze deep RNA sequencing data from WT and Nudt21-cKO BMDMs. This analysis revealed that approximately 1,800 genes underwent Percent Distal PolyA Site Usage Index (PDUI) changes, 90% of which exhibited significant 3’UTR shortening (Fig. 5A). This finding underscores Nudt21’s conserved role in promoting 3’UTR lengthening across various cell types.

Given that Nudt21 loss might affect gene expression through mRNA 3′-UTR shortening [20], we further explored its impact on gene expression in activated macrophages. We identified 354 genes in Nudt21-cKO BMDMs whose PDUI and mRNA expression levels were altered by overlapping PUDI shortening and mRNA changes (Fig. 5B). GO analysis revealed that autophagy-related pathways were predominantly affected (Fig. 5C, Fig. S5F, G).

In terms of autophagy-related genes (Fig. 5D), RNA immunoprecipitation of IFNγ/LPS-treated WT BMDMs with an Nudt21 antibody confirmed the direct binding of Nudt21 to the mRNAs of Map1lc3b and Ulk2 but not Wipi2 (Fig. 5E). *Map1lc3b* (LC3B) and *Ulk2* are essential for autophagosome formation and initiation, with LC3B involved in the expansion and closure of the autophagosome membrane and Ulk2 acting as an upstream initiator of autophagy by phosphorylating various downstream targets [47]. Significant 3’UTR shortening in *Map1lc3b* and *Ulk2* was observed in Nudt21-cKO BMDMs (Fig. 5F), as validated by RT‒qPCR via primers targeting both proximal and distal PAS (Fig. 5G, H). 3’ UTRs contain destabilizing elements, such as AU-rich elements (AREs), GU-rich elements (GREs), and PUF protein-binding sites, which regulate mRNA stability through interactions with RNA-binding proteins (RBPs) and miRNAs [16, 20, 48]. The inclusion or exclusion of these elements due to 3’ UTR-APA can substantially affect transcript stability. To investigate whether 3’ UTR shortening influences mRNA stability, we measured the decay rates of the *Map1lc3b* (NM_026160) and *Ulk2* (NM_013881) mRNAs at different time points following actinomycin D treatment. RT‒qPCR analysis revealed delayed mRNA decay in Nudt21-cKO BMDMs compared with WT BMDMs, whereas Wipi2 mRNA decay rates remained unaffected (Fig. 5I, J, Fig. S5H).

Together, these results demonstrate that Nudt21 represses the mRNA stability of the critical autophagy regulators *Map1lc3b* and *Ulk2* by directing the selection of their 3’UTR polyA sites, contributing to the regulation of autophagy and inflammatory responses in macrophages.

### Nudt21 impedes autophagy’s role in reducing inflammatory cytokine production

**Fig. 6:**
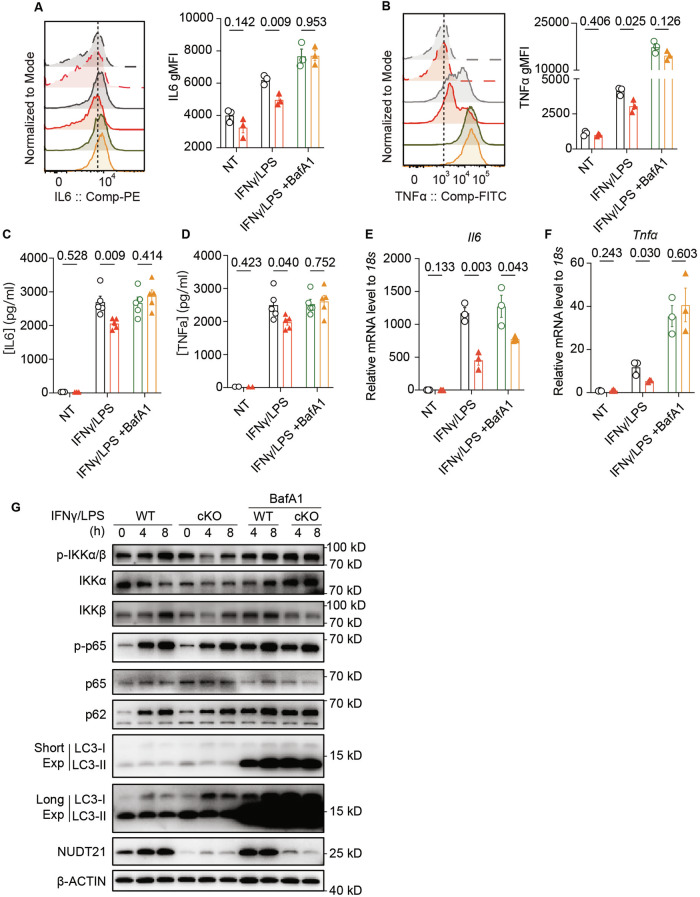
Nudt21 Impedes Autophagy’s Role in Reducing Inflammatory Cytokine Production. **A**, **B** Histograms displaying cytokine levels in BMDMs from WT and Nudt21-cKO mice primed with 50 ng/ml IFNγ overnight and 50 ng/ml LPS for 8 hours (*n* = 3 per group): (**A**) IL6 gMFI. (**B**) TNFα gMFI. ELISA results showing the IL6 (**C**) and TNFα (**D**) levels in the supernatants of the cultures primed with 50 ng/ml IFNγ overnight and 50 ng/ml LPS for 12 hours, with or without 100 nM BafA1. (*n* = 5 per group). RT‒qPCR analysis of cytokine mRNA levels in BMDMs primed with 50 ng/ml IFNγ overnight and 50 ng/ml LPS for 8 hours: (**E**) IL6 mRNA levels (n = 3 per group). (**F**) TNFα mRNA levels (*n* = 3 per group). **G** Immunoblot analysis of phosphorylated IKKα/β, total IKKα, total IKKβ, phosphorylated NF-κB p65 (p-p65), total p65, p62, LC3, and NUDT21 levels in WT and Nudt21-cKO BMDMs primed with 50 ng/ml IFNγ overnight and 50 ng/ml LPS, with or without 100 nM BafA1, for the indicated durations. β-ACTIN was used as the loading control. The error bars represent the standard errors of the means (SEMs). P values were determined by unpaired Student’s *t*-test

Recent research highlights macrophage autophagy as a pivotal anti-inflammatory mechanism across various inflammatory diseases [40, 42, 43, 49]. Given these findings, we hypothesized that the absence of Nudt21 might enhance autophagic flux in macrophages, thereby reducing inflammatory responses. To test this hypothesis, we treated BMDMs with the lysosomal inhibitor bafilomycin A1 (BafA1) during IFNγ/LPS stimulation. The flow cytometry results revealed that BafA1 attenuated the differences in the IL6 and TNFα protein levels between the wild-type and Nudt21-cKO BMDMs (Fig. 6A, B). This result was corroborated by ELISA, which revealed similar cytokine levels in both groups upon BafA1 treatment (Fig. 6C, D). RT‒qPCR analysis revealed that the differences in *Il6* and *Tnfα* mRNA levels between the two groups were significantly reduced after BafA1 treatment (Fig. 6E, F).

Considering the established connection between autophagy and the NF-κB signaling pathway [43, 50–53], we further examined changes in NF-κB activation. Compared with WT BMDMs, Nudt21-cKO BMDMs presented decreased phosphorylation levels of NF-κB. Additionally, the expression level of p62 (Sqstm1), a canonical autophagy adaptor, increased over time in WT BMDMs but decreased in Nudt21-cKO BMDMs. Following BafA1 treatment, there was a significant increase in NF-κB phosphorylation in Nudt21-cKO BMDMs, similar to the changes observed in p62 and LC3B levels (Fig. 6G). Moreover, we detected reduced phosphorylation and total levels of both IKKα and IKKβ in the Nudt21-cKO group, indicating impaired activation of these kinases. Interestingly, when the autophagy pathway was inhibited by BafA1, we observed a marked increase in IKKα levels in the cKO group, whereas IKKβ levels remained unchanged (Fig. 6G). These results suggest that Nudt21 deficiency may enhance the autophagic degradation of IKKα, which in turn could disrupt the IKKα‒NF-κB p65 axis, potentially leading to altered macrophage inflammatory responses.

Taken together, these findings suggest a critical role for Nudt21 in impeding autophagic flux in macrophages and its subsequent augmentation of inflammatory responses. This regulatory mechanism underscores the importance of Nudt21 in controlling inflammatory diseases, highlighting its potential as a therapeutic target for conditions characterized by dysregulated autophagy and inflammation.

## Discussion

Our study elucidates a novel RNA metabolism mechanism in which Nudt21-mediated alterations in 3’UTR length control autophagy activity in monocyte-derived macrophages during inflammation. While previous research has demonstrated the protective role of autophagy genes in macrophages during systemic hyperinflammation [41, 54–57], the specific upstream molecular mechanisms, particularly those involving RNA metabolism, remain poorly understood. Furthermore, despite advances in understanding mRNA APA events in activated macrophages and recent discoveries regarding the role of CPSF6 during viral infections [17–19, 58, 59], substantial gaps exist in our knowledge of APA regulators such as Nudt21 in macrophage-mediated inflammatory diseases.

We discovered that the absence of Nudt21 leads to sustained autophagy activation in macrophages, resulting in diminished inflammatory responses. Specifically, Nudt21 binds directly to the mRNAs of critical autophagy modulators, Map1lc3b and Ulk2, to restrain autophagic flux and augment the inflammatory response in a hyperinflammatory disease mouse model. Mechanistically, Nudt21 deletion results in mRNA 3’UTR shortening and increased mRNA stability of Map1lc3b and Ulk2, increasing autophagic flux, thereby decreasing NF-κB signaling and attenuating inflammatory cytokine secretion upon macrophage activation following IFNγ/LPS stimulation (Fig. S6).

The relatively low neutrophil infiltration observed in the colon of DSS-treated mice (Fig. S2D–F) may be attributed to the specific timing and progression of the inflammatory response at harvest. Early recruitment of neutrophils can occur but may not be sustained, resulting in limited tissue accumulation at later time points. Additionally, regulatory pathways or anti-inflammatory mediators activated as inflammation progresses may further restrict neutrophil migration, modulating the overall inflammatory response.

The influence of 3’UTRs on autophagy-related genes (ATGs) warrants further investigation, particularly to identify potential additional targets regulated by Nudt21 beyond Map1lc3b and Ulk2. Besides, we noted altered mitochondrial fitness (Fig. S5K) and cell viability (Fig. S3H-K) in Nudt21-deficient macrophages, suggesting broader roles for Nudt21 that extend beyond autophagy regulation, potentially impacting mitochondrial function and, consequently, oxidative stress and inflammation.

Given the intricate links between autophagy and inflammatory signaling pathways, more research is necessary to fully understand the extensive effects of Nudt21-mediated alterations in autophagy beyond the NF-κB pathway explored in this study. Furthermore, considering that 3’UTR length can be influenced by various RNA-binding proteins within the APA machinery and other RNA processing events, it is plausible that additional regulators collaborate with Nudt21 to influence autophagy through mechanisms that affect the RNA metabolism of ATGs.

Notably, alternatively spliced isoforms of autophagy components have implications in diseases such as cancer and neurological disorders [60]. Dysregulation of pre-mRNA processing involving key ATG genes is linked to cancer progression [61–64], whereas splice mutations in genes such as PINK1 and PRKN are associated with Alzheimer’s and Parkinson’s diseases [65–68]. These insights underscore the need for further studies to explore the potential contributions of these mutations to immune-mediated inflammatory diseases and to develop therapeutic strategies that leverage our understanding of APA regulators.

Several studies underscore the role of APA in inflammation, with 3′UTR-APA alterations implicated in diseases such as diabetic nephropathy and viral infection-induced inflammation [69, 70]. These findings demonstrate the importance of APA in regulating inflammatory gene expression and highlight the necessity of further exploration across different disease contexts. However, the complexity and cell-type specificity of APA remain significant challenges, as diverse patterns across cell types and disease states complicate generalization. Moreover, APA interacts with other RNA processes, such as splicing and stability, further complicating the identification of direct effects of APA on inflammation. A notable advancement is the development of CPA-perturb-seq [71], which enables precise perturbation of APA events via single-cell RNA sequencing. This approach allows for multiplexed screening and high-resolution analysis of APA-related gene alterations, offering new insights into the role of APA in inflammation and its potential as a therapeutic target.

In conclusion, our findings reveal that Nudt21-mediated alternative polyadenylation significantly modulates macrophage autophagy under hyperinflammatory conditions, highlighting the complex interplay between RNA metabolism and autophagy regulation. Given the role of dysregulated macrophage autophagy in disease pathogenesis, targeting the 3’UTRs of autophagy genes has emerged as a promising therapeutic approach to modulate specific autophagy pathways. This strategy offers the potential for precise anti-inflammatory treatments without compromising overall macrophage functionality, presenting exciting prospects for clinical application.

## Materials and methods

### Mice

*Nudt21-floxed* mice were generated at Cyagen Biosciences, Inc., by inserting two loxP sites into the loci flanking exons 2~3.

LysM^cre^ mice were crossed with *Nudt21*^*flox/flox*^ mice to produce myeloid–conditional Nudt21 knockout mice (*Nudt21*^*fl/fl*^ LysM^Cre^). The sex-matched LysM^Cre^ littermates were considered wild-type (WT) controls. All the mice used in this study were of the C57BL/6 background, maintained in specific pathogen–free facilities and used according to protocols approved by the Animal Care and Use Committees of the Shanghai Jiao Tong University School of Medicine.

PCR genotyping of *Nudt21* tail genomic DNA with primers.

5’- GAAAACTACCAAAACGTCAGGAGA -3’ and 5’- TAGAGTCAACCAATCCAGCTGAC -3’

### Primary cell isolation

The spleen was collected and pressed through a 200-gauge mesh. Spleen cells were prepared by lysing erythrocytes with red blood cell lysis buffer (Thermo Fisher Scientific).

Peritoneal exudate cells were harvested via peritoneal lavage using ice-cold HBSS (Thermo Fisher Scientific) supplemented with 2% fetal bovine serum (FBS, HyClone).

For isolation of colon lymphocytes, the colon was cut and flushed with ice-cold phosphate-buffered saline (PBS) and then cut into 1-cm-long pieces and incubated in extraction buffer (2% FBS, 2 mM EDTA, and 1 mM dithiothreitol in PBS) with shaking (200 rpm) at 37°P for 30 min. After extensive washing to remove intraepithelial lymphocytes, the lamina propria was minced and incubated at 37°C with shaking (200 rpm) for 60 min in digestion buffer (RPMI 1640 with 2% FBS, type II collagenase (1 mg/ml, catalog no. 17101015, Gibco), and dispase (0.5 mg/ml, catalog no. D4693, Sigma)). The supernatant was collected, and the lymphocytes were purified via Percoll (catalog no. 17089109, GE Healthcare) gradient centrifugation.

### Flow cytometry and cell sorting

For flow cytometry, 1 × 10^6^ cells were added to a 96-well V-bottom plate (Thermo Fisher Scientific) and incubated with 2.5 mg/ml blocking anti-CD16/32 antibody (catalog no. 101302, Biolegend) diluted in 1X DPBS supplemented with 2% FBS for 10 min at room temperature. After two washing steps, the cells were then processed for surface staining. For surface staining, the cells were stained for surface markers for 30 min at 4 °C. The cell pellet was resuspended in LIVE/DEAD stain containing PBS with 2% FBS. For intracellular cytokine staining, the cells were restimulated for 3 h at 37 °C with GolgiPlug (1 μl ml^−1^, catalog no. 555029, BD Biosciences). After stimulation, the cells were washed and stained according to the manufacturer’s protocol (catalog no. 554714, BD Biosciences). To stain nuclear factors, the cells were fixed and stained according to the manufacturer’s instructions (catalog no. 00--5523--00; eBioscience). The antibodies used for flow cytometry are listed in the table. S1.

For bone marrow monocyte (BMM) cell sorting, bone marrow cells from WT and Nudt21-cKO mice were stained with the indicated surface markers, and CD45.2 APC-CY7, CD11b BV650, Ly6C FITC, Ly6G PE fluorescence-conjugated antibodies and Zombie Aqua (L/D) (catalog no. 423102, Biolegend), L/D^−^CD45.2^+^CD11b^+^Ly6G^−^Ly6C^+^ cells were gated as the BMM cell population.

Stained cells were either acquired on an LSRFortessa X-20 or LSRFortessa (BD Biosciences) or sorted on a FACSAria III (BD Biosciences) by the Flow Core Facility of Shanghai Institute of Immunology, Shanghai Jiao Tong University School of Medicine. Flow cytometry data were analyzed via FlowJo software (v.10.0 or higher, FlowJo LLC).

### DSS-induced colitis model

WT and Nudt21-cKO mice were treated with 3% DSS [26–28] (catalog no. 60316ES76, Yeasen) in the drinking water for 5 days, followed by access to regular drinking water for 2 days. Body weight was measured daily, and the mice were euthanized on day 8. Colon length was measured. Colones were fixed with 4% paraformaldehyde for histology or used for the isolation of colonic immune cells as described above. Mice of the same sex were age-matched for each experiment and were aged between 10 and 12 weeks.

### PolyI:C- and LPS-induced murine HLH

Hemophagocytic lymphohistiocytosis (HLH) was induced by sequential challenge with polyI:C and LPS as previously described (Wang et al., [29]). The mice were injected intraperitoneally with high-molecular-weight polyI:C (catalog no. tlrl-pic, InvivoGen) at 10 mg/kg body weight reconstituted in PBS. Twenty-four hours after polyI:C injection, the mice were injected intraperitoneally with LPS (catalog no. l2880, Sigma) at 5 mg/kg body weight reconstituted in PBS. To induce systemic macrophage depletion, 200 μL of clodronate liposomes (catalog no. 40337ES08, Yeasen) were injected into the venous sinuses of the mice. The mice in the control group received an equivalent volume of control liposomes (PBS) (catalog no. 40338ES08, Yeasen). Mice of the same sex were age-matched for each experiment and aged 8--10 weeks old.

### Histological analysis

Tissues from the proximal colon were dissected and fixed with 4% paraformaldehyde. The tissues were then embedded in paraffin, sectioned at 5 µm, and stained with H&E. The sections were then blindly analyzed via light microscopy (Olympus) and scored according to a scoring system referred to in the Histopathological Atlas of New Drug Toxicological Laboratory Animals.

### Bone marrow-derived macrophages (BMDMs)

Bone marrow-derived macrophages were prepared from bone marrow cells harvested from hind leg femurs. The cells were cultured in 10-cm nontreated dishes for 5‒6 days (37°C, 10% CO_2_) in 15 mL of RPMI 1640 medium (Gibco) supplemented with 10% fetal bovine serum (FBS, HyClone), 50 U/mL penicillin and 50 mg/mL streptomycin and supplemented with 100 ng/ml M-CSF (catalog. 576408, Biolegend). An additional 10 mL of culture medium was added on day 2. Differentiated BMDMs were harvested and replated in sterile 24-well nontreated tissue culture plates at 5×105 cells/well in a final volume of 500 µL of 1640/FBS supplemented with 100 ng/ml M-CSF for 12 hours. For proinflammatory activation, plated BMDMs were primed with IFNγ (50 ng/mL, catalog no. 315-05-1 mg, PeproTech), followed by LPS (50 ng/mL, catalog no. l2880, Sigma).

### RNA-seq

Total RNA was extracted from the BMDMs of WT and Nudt21-cKO mice, either with or without IFNγ (50 ng/ml) treatment overnight, followed by LPS (50 ng/ml) stimulation for 4 hours via TRIZOL (catalog no. 15596026; Invitrogen). For each sample, BMDMs from two mice were pooled, and two samples from WT and two from cKO mice were prepared for RNA sequencing. RNA purification, reverse transcription, library construction, and sequencing were performed by Novogene Co., Ltd. Paired-end RNA-Seq was performed to yield more than 250 million reads per sample, which allowed us to obtain not only transcript expression but also alternative PAS usage. Briefly, RNA purity was checked via a NanoPhotometer® spectrophotometer (IMPLEN, CA, USA), and RNA integrity was evaluated via the RNA Nano 6000 Assay Kit of the Bioanalyzer 2100 system (Agilent Technologies, CA, USA). The sequencing libraries were generated via the NEBNext Ultra RNA Library Prep Kit for Illumina (NEB, USA, Catalog #: E7530L) following the manufacturer’s recommendations, and index codes were added to attribute sequences to each sample. The qualified libraries were pooled and sequenced on Illumina platforms via the PE150 strategy at Novogene Bioinformatics Technology Co., Ltd. (Beijing, China).

The clean reads were aligned to the mouse genome (mm10, GRCm38) via HISAT2 2.2.1 (Kim *et al*., 2015), and RefSeq gene expression was quantified via Stringtie 2.1.7 (Pertea et al., 2015). Significant genes were defined by an absolute fold change > 1.5 and a *p*-value < 0.05.

### Analysis of APA from RNA-seq

The well-established algorithm DaPars [46] (https://github.com/ZhengXia/DaPars) was used to calculate the percentage of distal PAS usage index (PDUI) for each gene (Xia et al., [46]). APA events with *p* values < 0.05 and PDUI changes > 0.1 were considered statistically significant for the comparison between WT and Nudt21-cKO BMDMs.

### RT‒qPCR

For quantitative real-time PCR, total cellular RNA or RNA in the polysome fraction was extracted via TRIZOL (catalog no. 15596026; Invitrogen). mRNA was reverse transcribed into cDNA via HiScript III RT SuperMix for qPCR (+gDNA wiper) (catalog no. R323-01, Vazyme). Fluorescence real-time PCR was performed with the ChamQ Universal SYBR qPCR Master Mix (catalog no. q711-03, Vazyme) with the CFX384 Touch Real-Time PCR Detection System (Bio-Rad, USA). The primers used are listed in Table S1.

### Distal polyadenylation site usage assay

RT‒qPCR was performed to investigate distal polyadenylation site (dPAS) usage in selected genes. The common primer was designed to target the open reading frame and was used for detecting the total transcript level. The distal primer was designed to target sequences just before the dPASs and detect long transcripts. The relative dPAS usage was represented by the ratio of long transcripts to total transcripts. The primers used are listed in Table S2.

### RNA immunoprecipitation (RIP) assay

The RIP assay was performed as described previously [72]. BMDMs isolated from WT mice were lysed in RIP lysis buffer (150 mM NaCl, 10 mM Tris, 0.1% NP-40, pH 7.4, supplemented with RNase inhibitor) for 3 h at 4 °C. Then, 100 µl of whole-cell extract was incubated with Dynabeads™ Protein G (catalog no. 1004D; Thermo Fisher Scientific) conjugated with anti-Nudt21 (1:100 dilution; catalog no. 10322-1-AP; Proteintech) or IgG (1:100 dilution; catalog no. 2729S; Cell Signaling Technology) at 4 °C for 6 h. Thereafter, the beads were incubated with proteinase K with shaking to remove protein. The coprecipitated RNAs were extracted via an RNeasy micro kit (catalog no. 74004, QIAGEN) and subjected to RT–qPCR as described above.

### RNA degradation assay

BMDMs were seeded on 12-well plates with 0.5 million cells per well. Actinomycin D (catalog no. HY-17559, MCE) was added at a final concentration of 5 μM. The cells were collected (after 0, 1, and 2 hours), and total RNA was extracted for real-time qPCR. The data were normalized to the *t* = 0 time point.

### Immunoblots

For western blot analysis, the cells were lysed in RIPA buffer (catalog no. P0013B, Beyotime) containing a protease and phosphatase inhibitor cocktail that was free of EDTA (catalog no. 78443, Thermo Fisher Scientific) for 30 min on ice, followed by pelleting insoluble material via centrifugation. The protein concentration was determined via a BCA assay.

### Immunofluorescence microscopy

The cells were fixed with 4% formaldehyde for 10 min at 37 °C, permeabilized with 0.25% Triton X-100 in PBS for 10 min and incubated for 1 h in PBS containing 5% BSA (blocking buffer) at room temperature. They were then incubated with primary antibodies against LC3B (1:250, catalog no. L7543, Sigma) diluted in blocking buffer overnight at 4 °C and then for 1 h with Alexa Fluor–conjugated secondary Abs (1:1000, catalog no. 4412, Cell Signaling Technology) and Lamp1-Cy3 (1:500, catalog no. ab67283, Abcam) in blocking buffer. DAPI Fluoromount-G™ (catalog no. 36308ES11, Yeasen) was used as the mounting medium. Images were acquired with an FV3000 confocal microscope (Olympus) and processed with Fiji (National Institutes of Health).

### TEM analysis

For morphological observation of autophagic vacuoles, BMDMs from WT and Nudt21-cKO mice were resuspended in 2.5% glutaraldehyde fixed solution overnight at 4 °C; images were captured via an Olympus EM208S transmission electron microscope (TEM) (Hitachi), Core Facility of the College of Basic Medical Sciences, Shanghai Jiao Tong University School of Medicine. TEM was adopted for morphology and elemental analysis on a JEOL JEM-2011 transmission electron microscope with an energy-dispersive X-ray spectroscopy detector operated at an accelerating voltage of 200 kV. Images were acquired via a Hitachi S4800 scanning electron microscope with a working voltage of 3 kV.

### Cytokine analysis

The plasma concentrations of IL-1β were measured via an IL-1β ELISA (catalog no. EK201B; Multi Sciences) according to the manufacturer’s instructions. IL6 and TNFα levels were measured via IL6 ELISA (catalog no. EK206, Multi Sciences) and TNFα ELISA (catalog no. EK282, Multi Sciences) according to the manufacturer’s instructions.

### Statistical analysis

Each data point from the graphs of the BMDM or mouse experiments represents an independent biological replicate (i.e., a different mouse). All in vivo experiments were conducted with at least two independent cohorts. All the data are presented as the means ± SEMs. Comparisons between groups were analyzed by the unpaired two-tailed Student’s t test or one-way analysis of variance (ANOVA). Survival curves were compared via the log-rank test. All graphical data were prepared and analyzed via GraphPad PRISM (version 10.1.1).

## Supplementary information

Source Imagaes of westernblots

Supplementary Figure 1

Supplementary Figure 2

Supplementary Figure 3

Supplementary Figure 4

Supplementary Figure 5

Supplementary Figure 6

Supplementary information

## Data Availability

All the data needed to evaluate the conclusions in this article are presented in the paper or the supplementary materials (or both). The bulk RNA-seq data generated in this study are available at the Gene Expression Omnibus (accession no. GSE267291). The Nutd21 flox mouse strain can be provided by H.-B.L. pending scientific review and a completed material transfer agreement. Requests for those mouse lines should be submitted to H.-B.L.
