# Supplementary material for: Alternative mRNA polyadenylation regulates macrophage hyperactivation via the autophagy pathway
Source: Cell Mol Immunol. 2024 Nov 13;21(12):1522–34. doi: 10.1038/s41423-024-01237-8 (PMC11607066; doi:10.1038/s41423-024-01237-8)

Source Fig. 4H

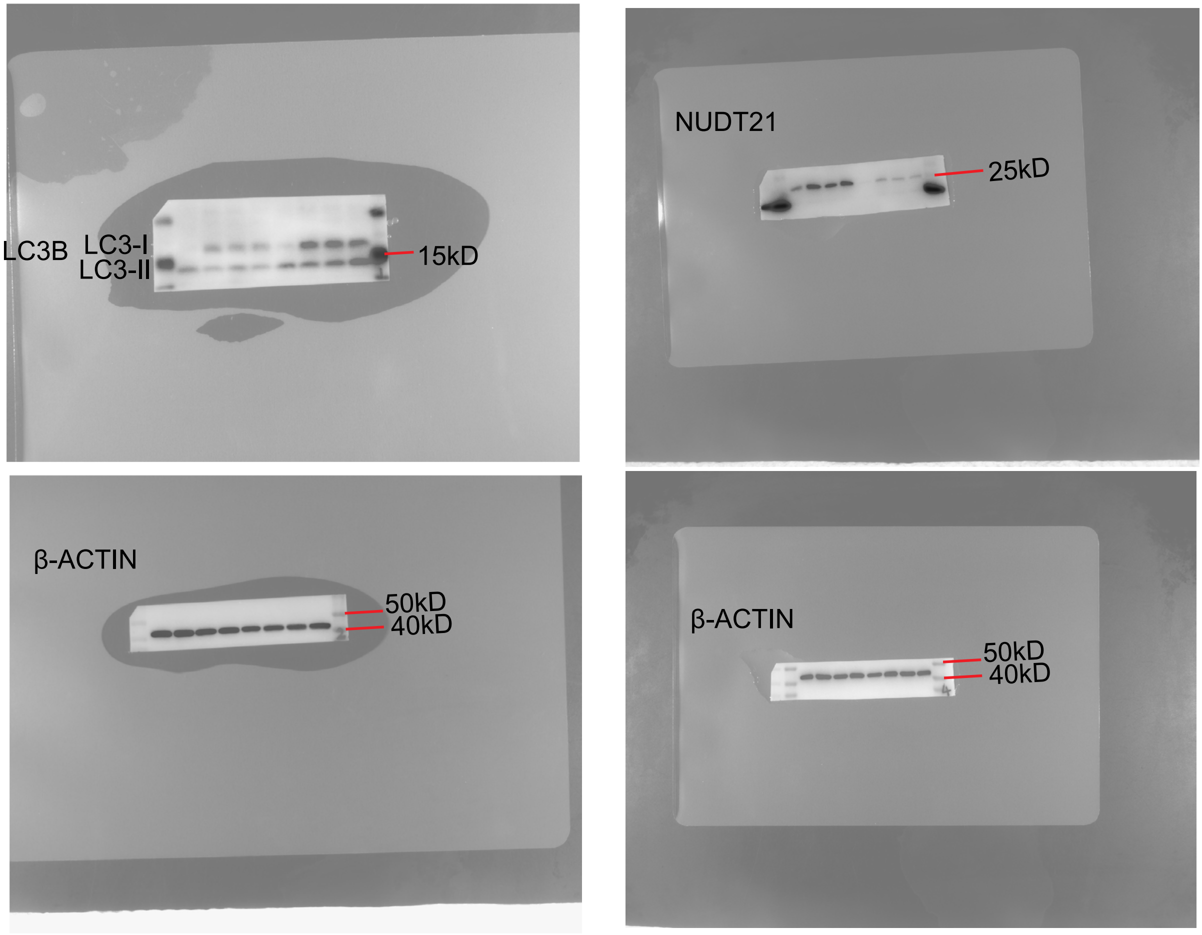

Source Fig. 6G

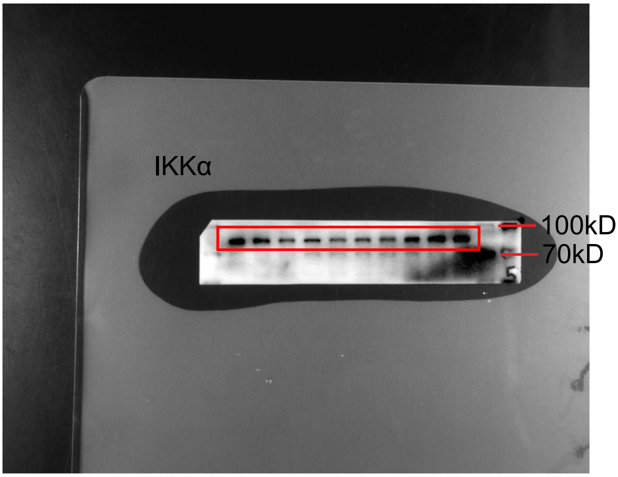

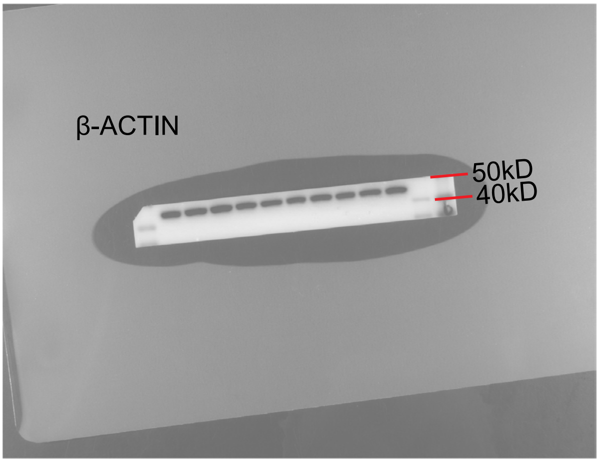

Source Fig. 6G

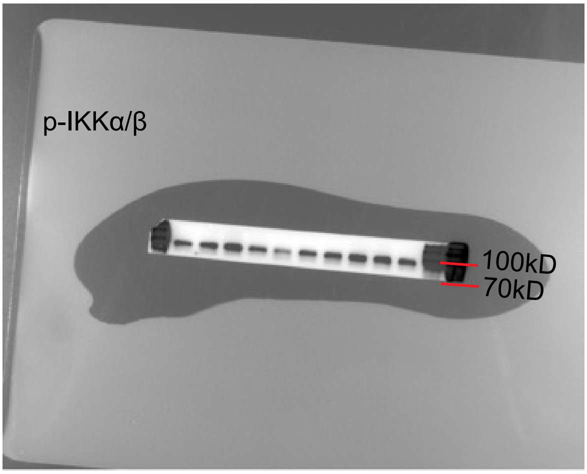

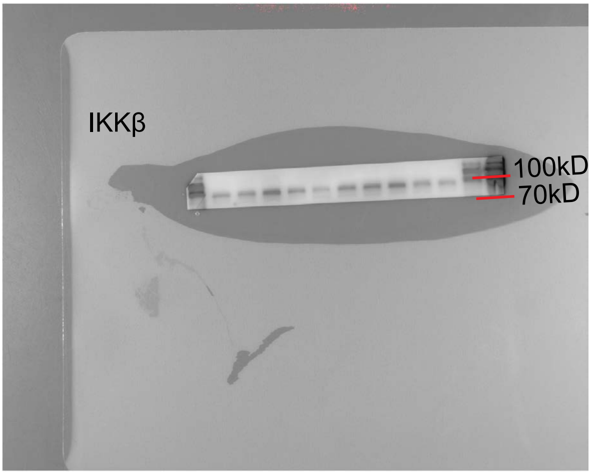

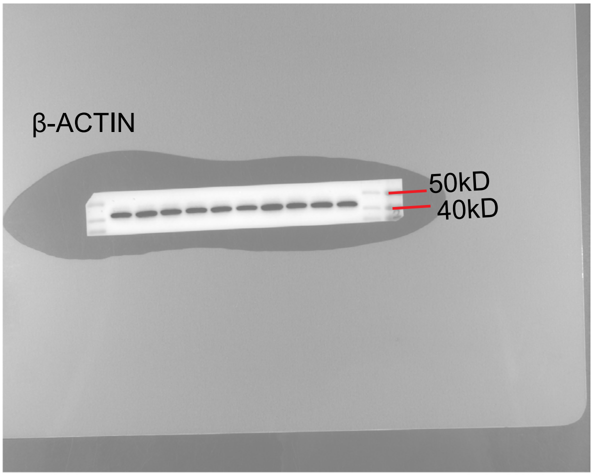

Source Fig. 6G

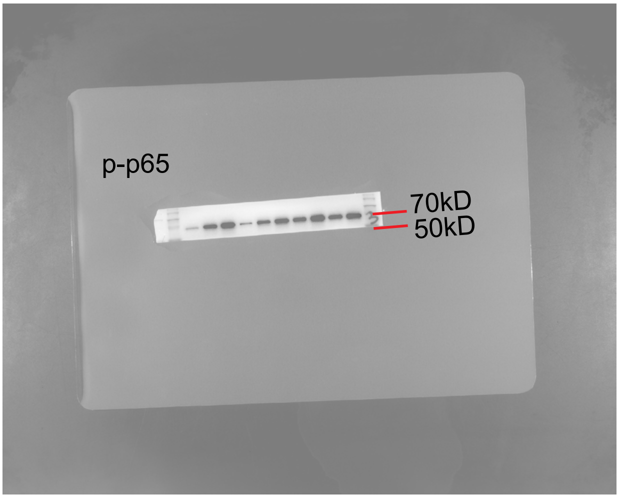

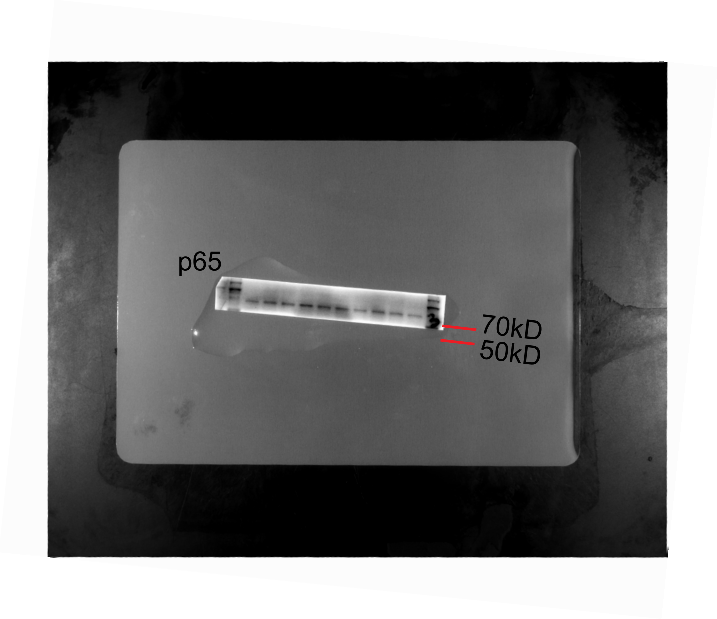

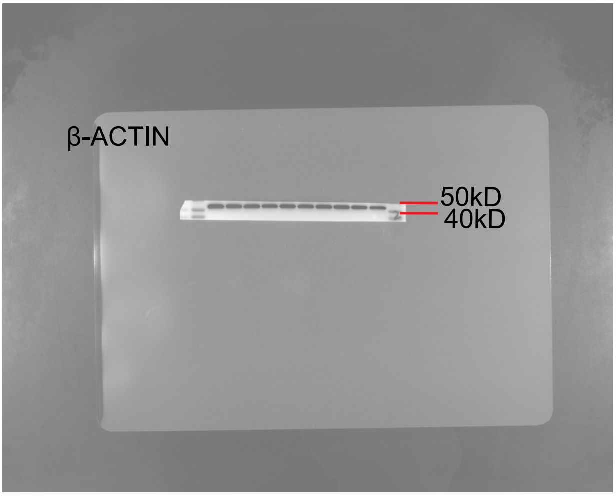

Source Fig. 6G

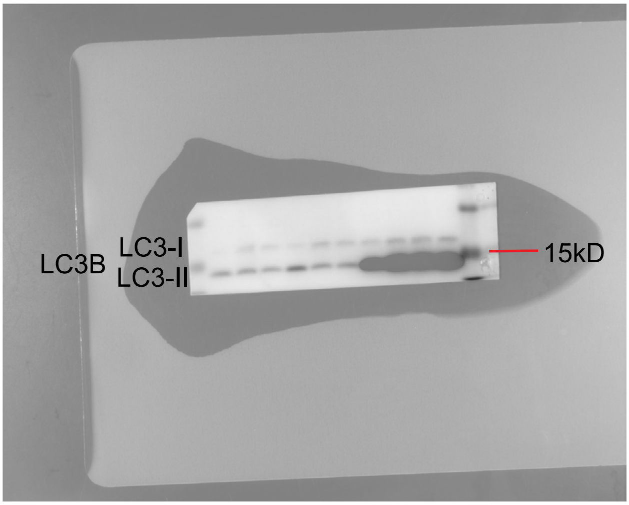

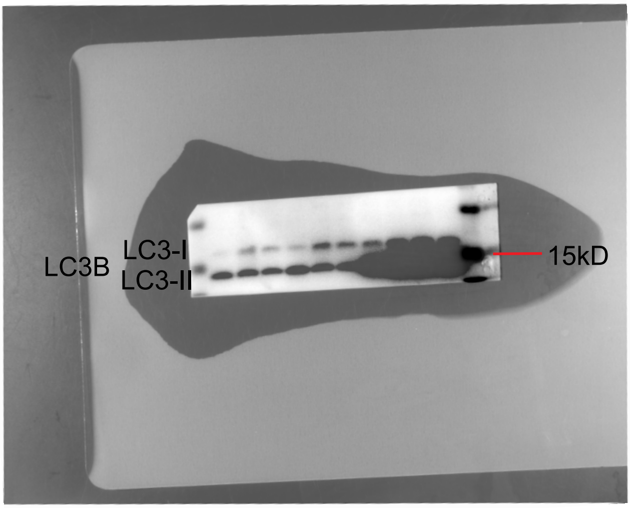

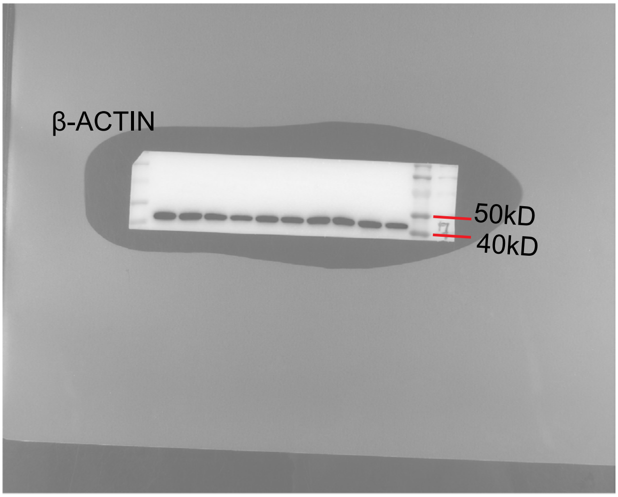

Source Fig. 6G

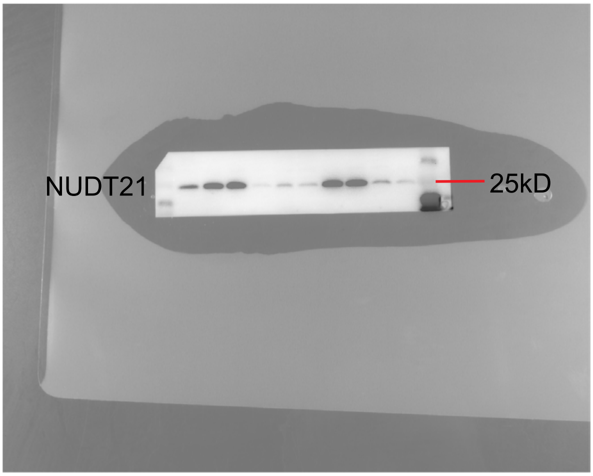

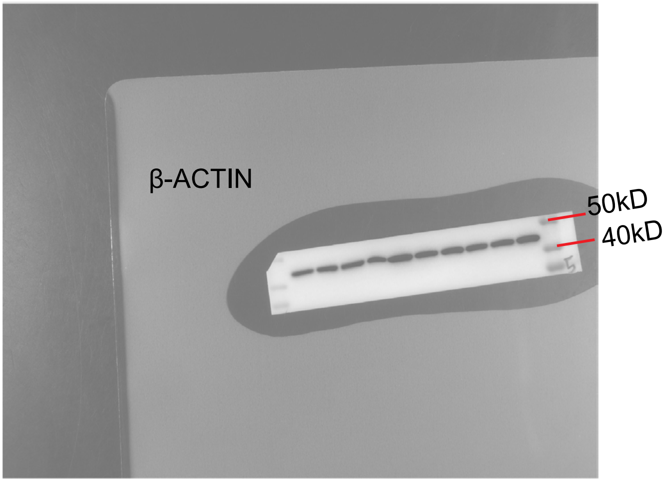

Source Fig. 6G

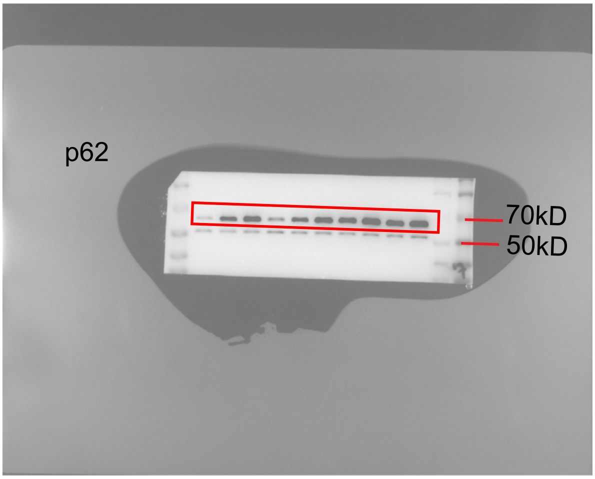

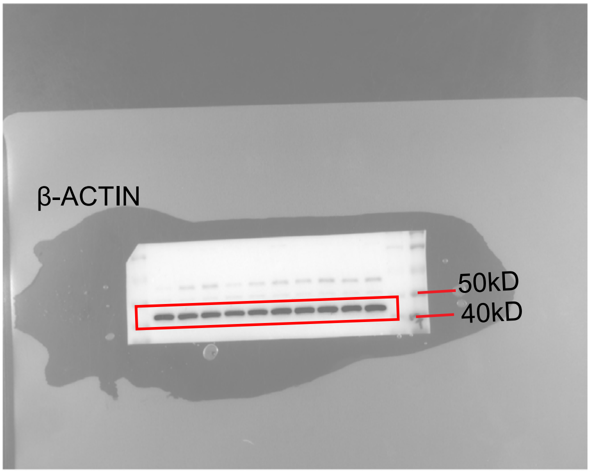

Source Fig. S1F

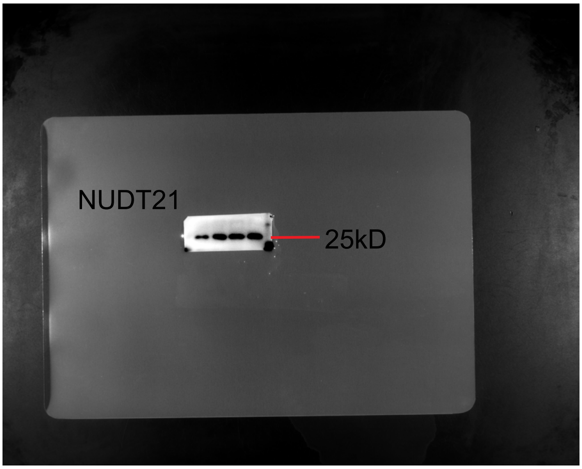

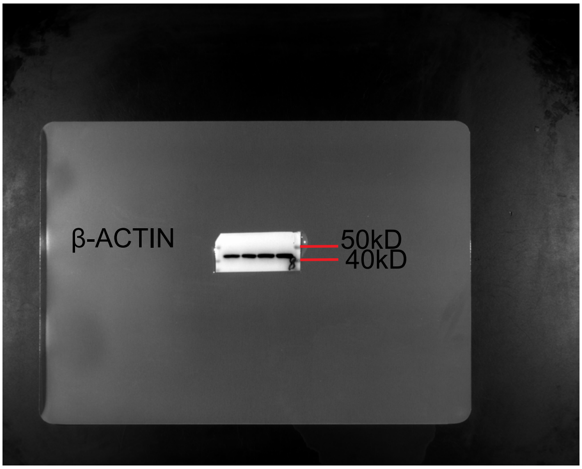

Source Fig. S2C

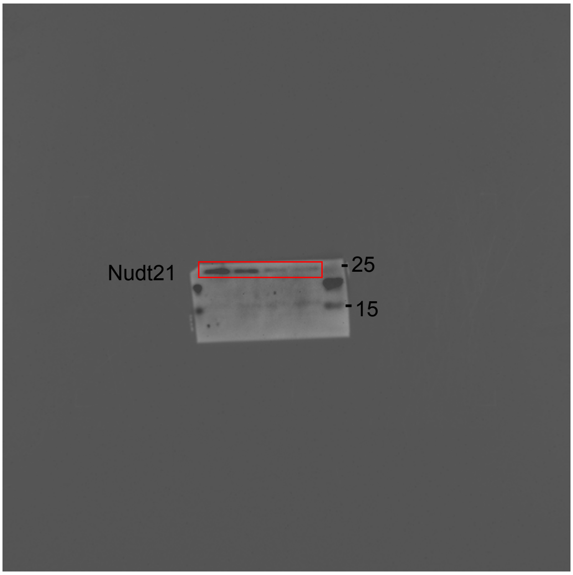

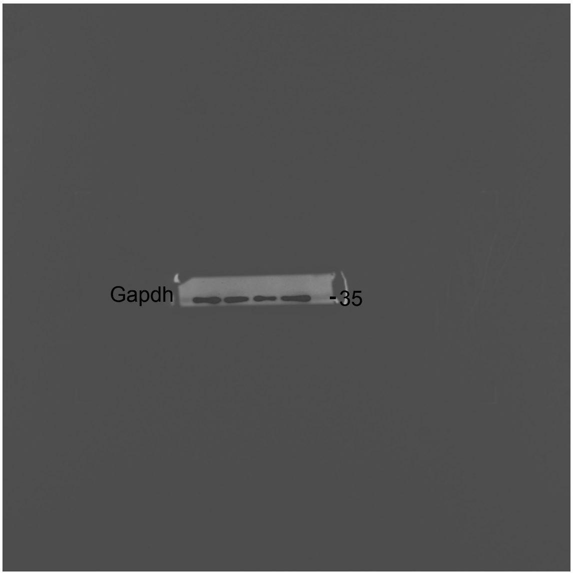

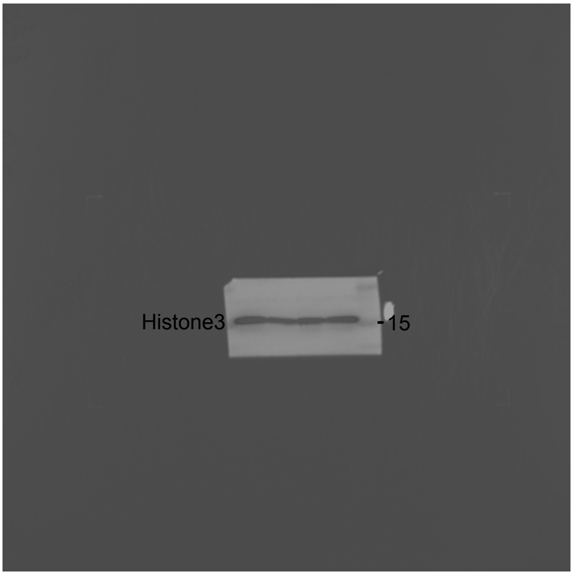

Supplement: Supplementary file 1 — Source Imagaes of westernblots [file 41423_2024_1237_MOESM1_ESM.pdf]
