# Supplementary figures and images for: Alternative mRNA polyadenylation regulates macrophage hyperactivation via the autophagy pathway
Source: Cell Mol Immunol. 2024 Nov 13;21(12):1522–34. doi: 10.1038/s41423-024-01237-8 (PMC11607066; doi:10.1038/s41423-024-01237-8)

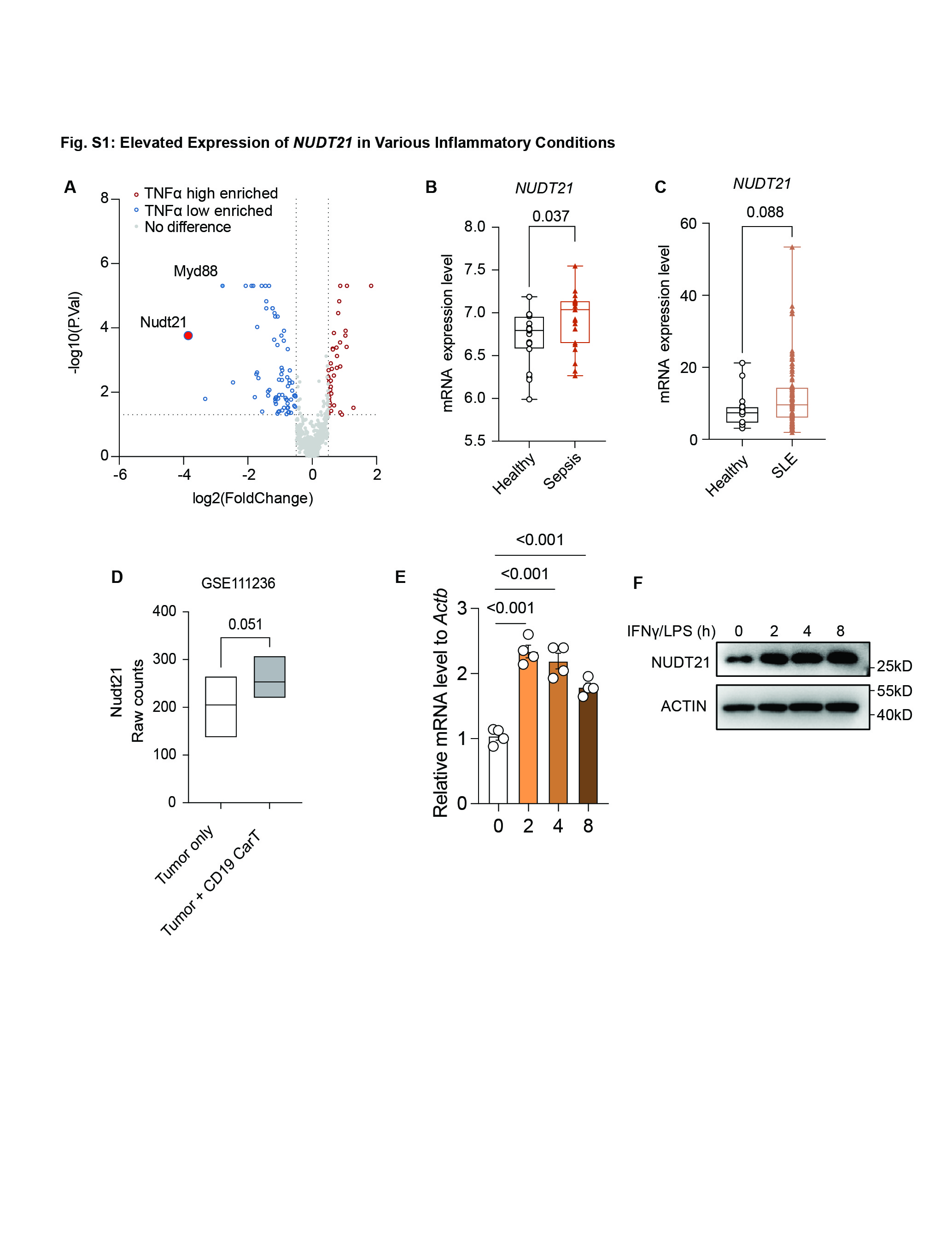

Supplement: Supplementary file 2 — Supplementary Figure 1 [file 41423_2024_1237_MOESM2_ESM.jpg]

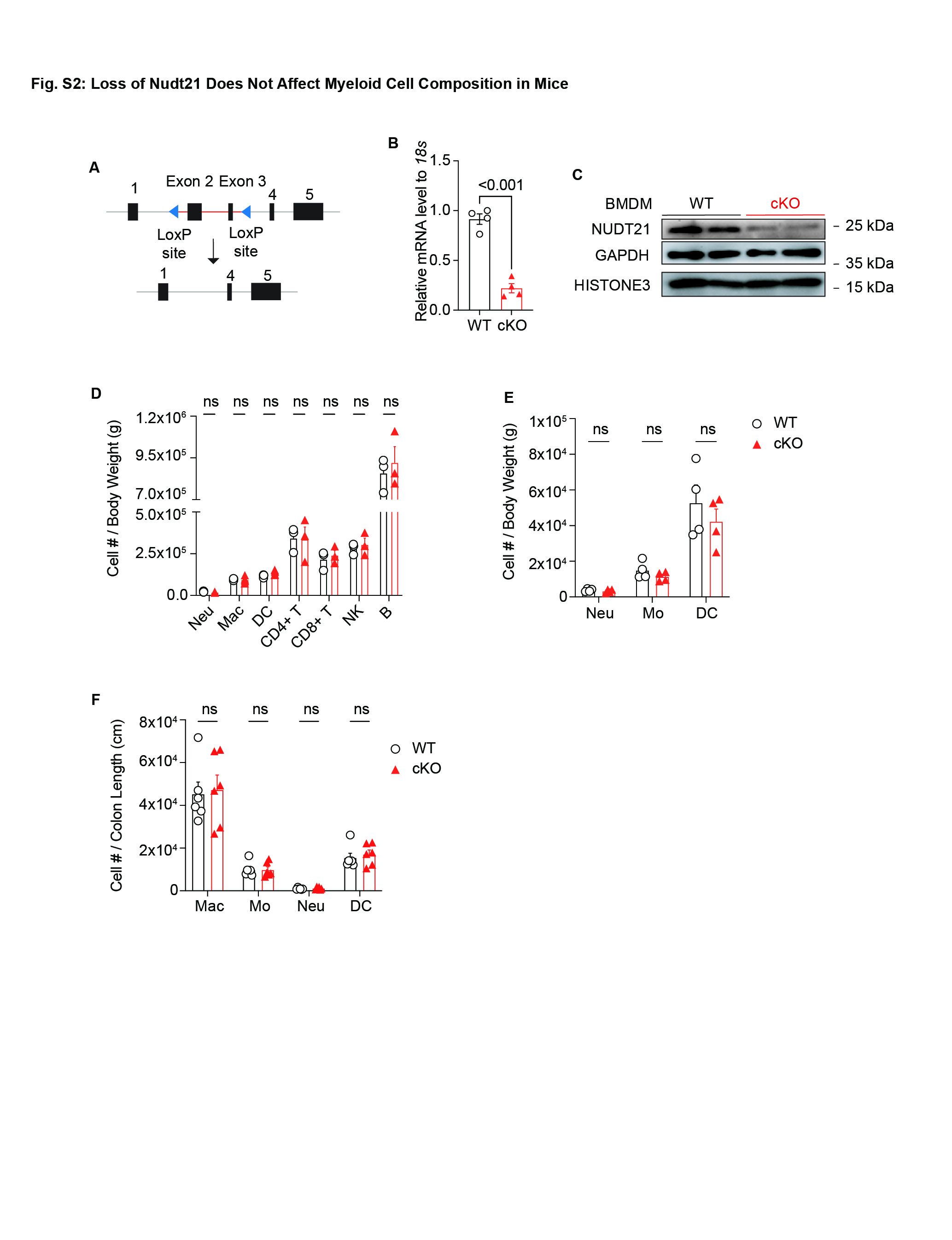

Supplement: Supplementary file 3 — Supplementary Figure 2 [file 41423_2024_1237_MOESM3_ESM.jpg]

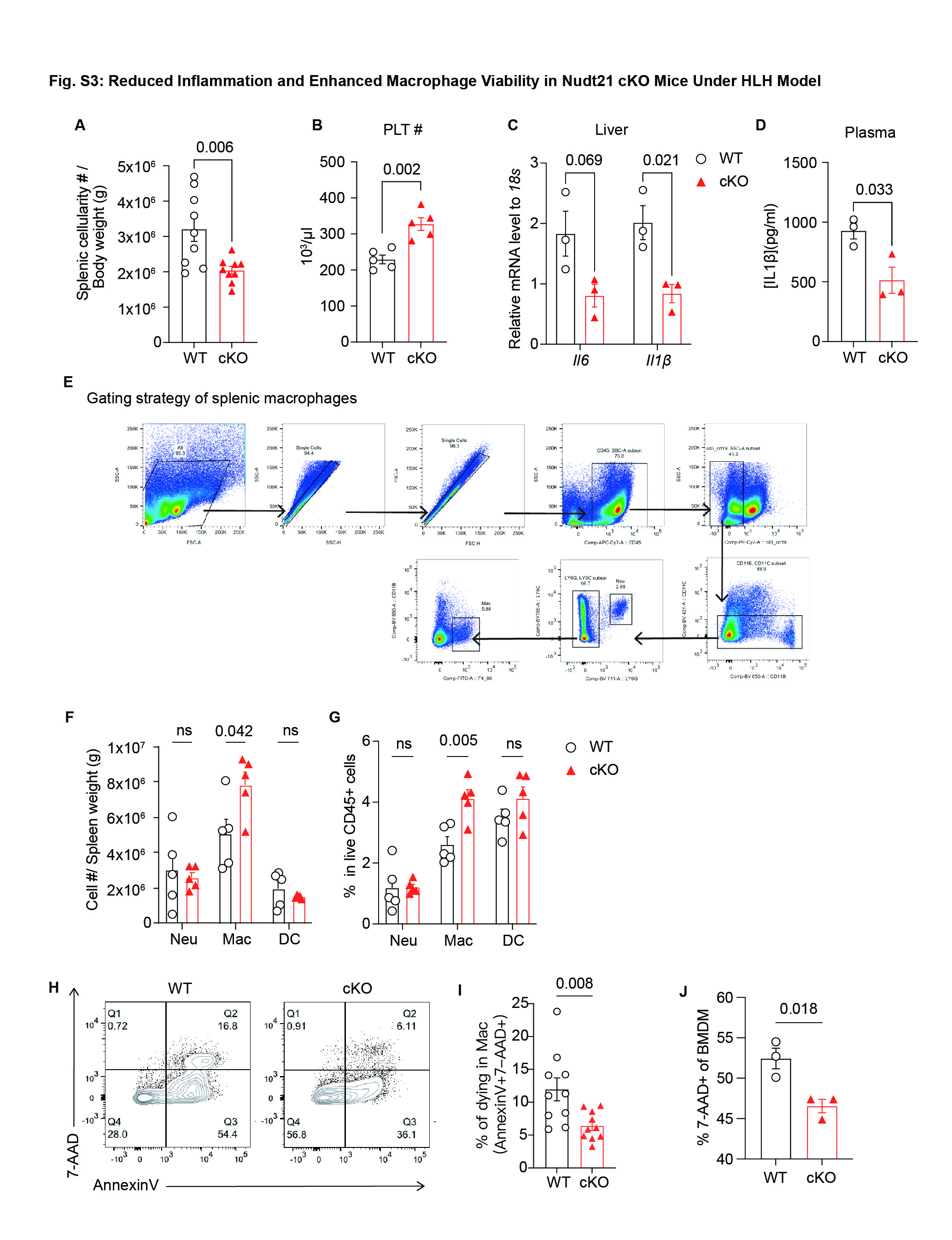

Supplement: Supplementary file 4 — Supplementary Figure 3 [file 41423_2024_1237_MOESM4_ESM.jpg]

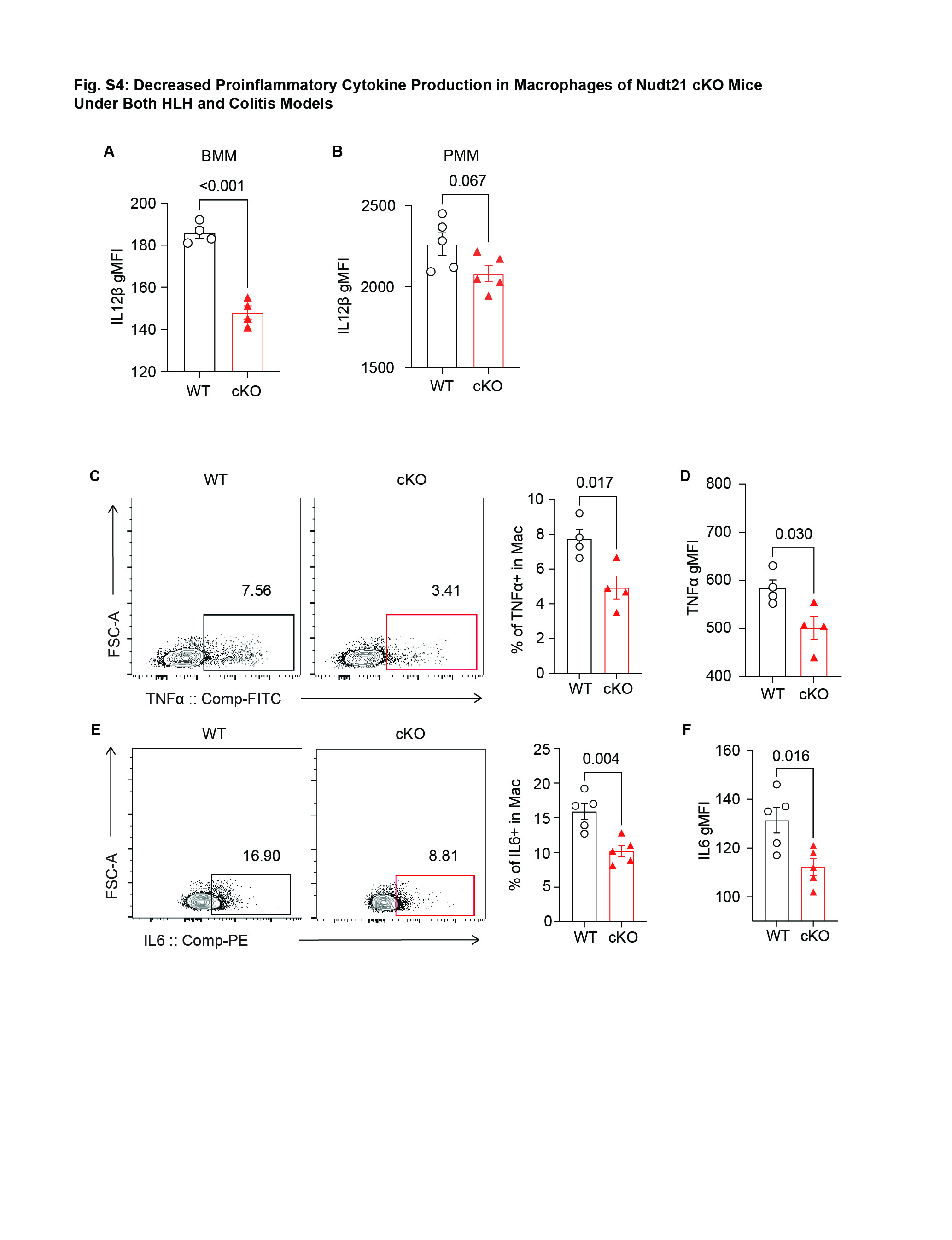

Supplement: Supplementary file 5 — Supplementary Figure 4 [file 41423_2024_1237_MOESM5_ESM.jpg]

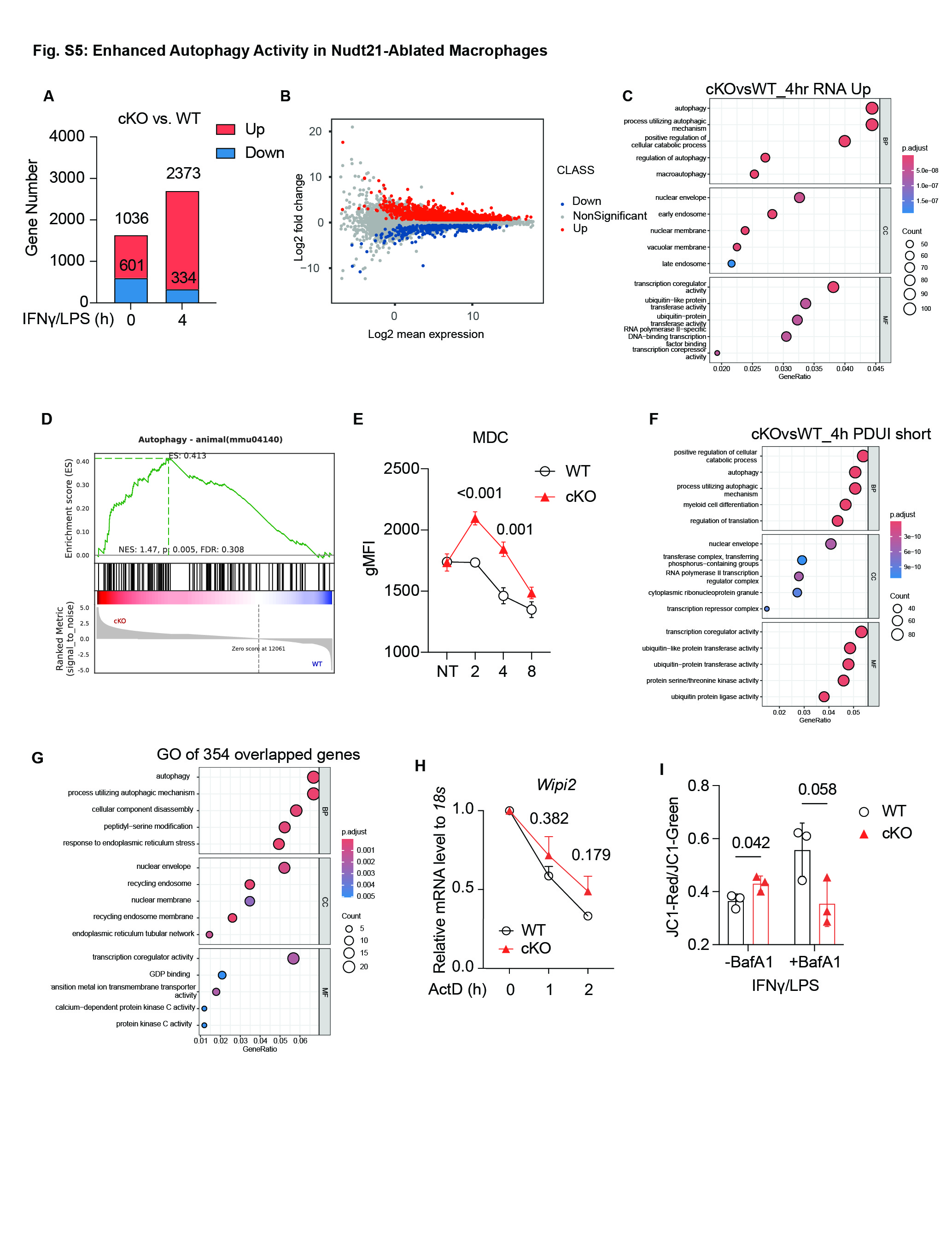

Supplement: Supplementary file 6 — Supplementary Figure 5 [file 41423_2024_1237_MOESM6_ESM.jpg]

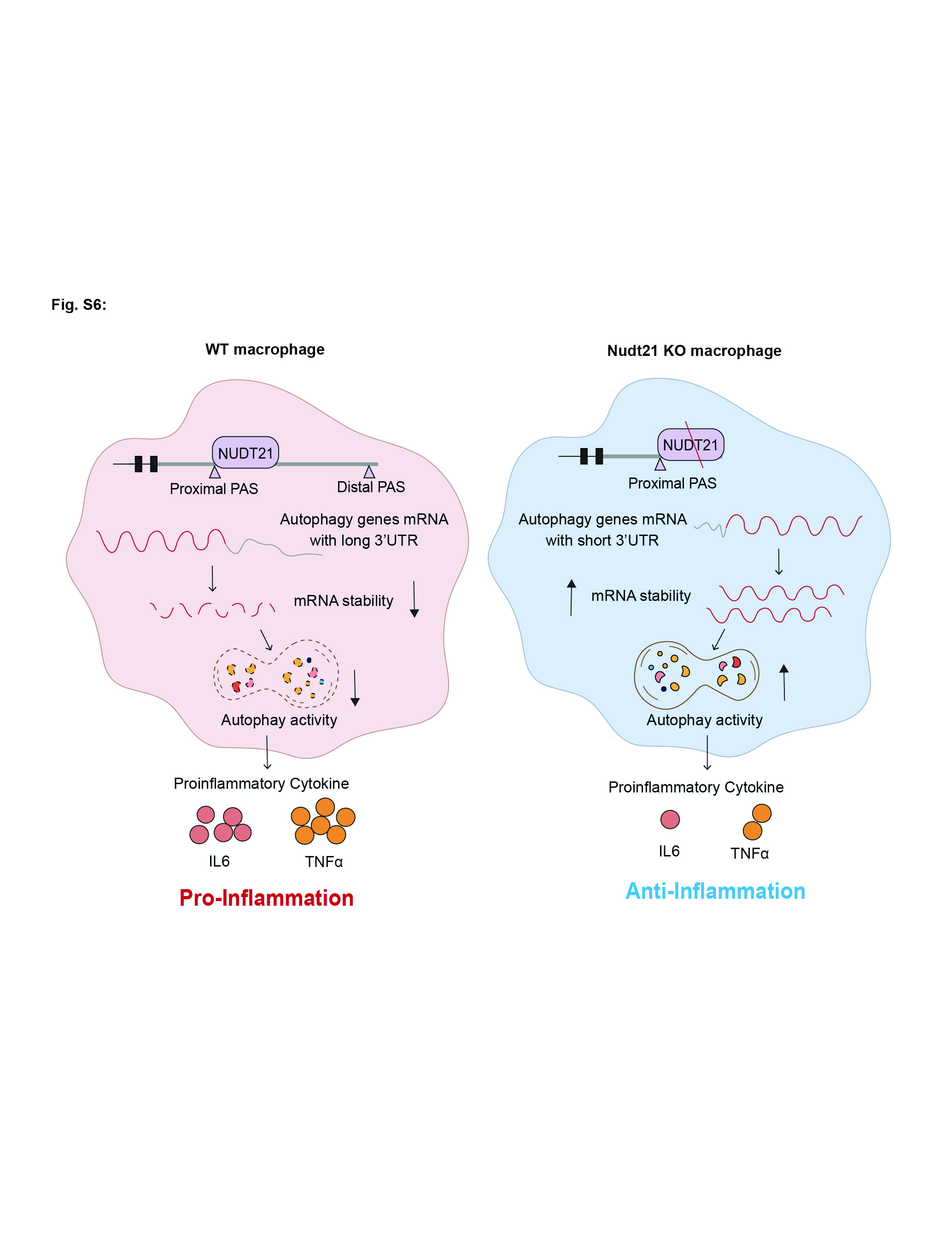

Supplement: Supplementary file 7 — Supplementary Figure 6 [file 41423_2024_1237_MOESM7_ESM.jpg]
