# Supplementary material for: Alternative mRNA polyadenylation regulates macrophage hyperactivation via the autophagy pathway
Source: Cell Mol Immunol. 2024 Nov 13;21(12):1522–34. doi: 10.1038/s41423-024-01237-8 (PMC11607066; doi:10.1038/s41423-024-01237-8)
Supplement: Supplementary file 8 — Supplementary information [file 41423_2024_1237_MOESM8_ESM.docx]

## Fig. S1: Increased expression of Nudt21 under various inflammatory conditions

**(A)** Volcano plot from the CRISPR screening results of Tong *et al*.^25^, which revealed that sgRNA-targeted genes were enriched in the TNFα-high (red) and TNFα-low (blue) populations. Notably, Nudt21 is highlighted with a large red dot.

**(B-D)** mRNA expression levels of NUDT21 in (B) blood samples from sepsis patients (GSE28750). (C) Systemic lupus erythematosus (SLE) patients (GSE72509). (D) Peritoneal monocyte-derived macrophages from CarT-induced cytokine syndrome mice (GSE111236).

**(E-F)** RT‒qPCR analysis (E) and immunoblot analysis (F) of Nudt21 in WT BMDMs primed with 50 ng/ml IFNγ overnight and stimulated with 50 ng/ml LPS for the indicated durations.

The error bars represent the standard errors of the means (SEMs). P values were determined by unpaired Student’s t test or one-way ANOVA.

## Fig. S2: Loss of Nudt21 does not affect myeloid cell composition in mice

**(A)** Schematic illustrating the generation of Nudt21-cKO mice.

**(B-C)** Analysis of Nudt21 knockout efficiency: (B) qRT‒PCR results showing Nudt21 expression in BMDMs from WT and Nudt21-cKO mice. (C) Immunoblot confirming the absence of Nudt21 protein in BMDMs from Nudt21-cKO mice.

**(D-E)** Histograms displaying the absolute number of immune cells per body weight (grams) analyzed. (D) The spleens of 8-week-old WT and Nudt21-cKO mice (n=3 per group) are shown. (E) Bone marrow of 8-week-old WT and Nudt21-cKO mice (n=4 per group).

**(F)** Histogram showing the absolute number of myeloid cells per colon length (cm) in the colons of 10-week-old WT and Nudt21-cKO mice in the colitis model (n=6 per group, data pooled from two separate experiments).

The error bars represent the standard errors of the means (SEMs). P values were determined by unpaired Student’s t test.

## Fig. S3: Reduced inflammation and enhanced macrophage viability in Nudt21-cKO mice in the HLH model

**(A)** Histograms displaying the absolute number of cells per body weight (g) in the spleens of WT and Nudt21-cKO mice analyzed at 6 hours post-LPS injection (n=9 per group, data pooled from two separate experiments).

**(B)** Absolute platelet counts in the peripheral blood of WT and Nudt21-cKO mice measured at 6 hours post-LPS injection (n=5 per group).

**(C)** RT‒qPCR analysis of *Il6* and *Il1β* mRNA levels in liver tissues from WT and Nudt21-cKO mice at 6 hours post-LPS injection (n=3 per group).

**(D)** Plasma IL-1β levels in WT and Nudt21-cKO mice were analyzed 6 hours after LPS injection (n=3 per group).

**(E)** Gating strategy for identifying splenic macrophages.

**(F)** Histograms showing the absolute number of myeloid cells per spleen weight (g) from WT and Nudt21-cKO mice analyzed at 6 hours post-LPS injection (n=5 per group).

**(G)** Histograms showing the percentage of specified myeloid cell populations within CD45+ cells from the spleens of WT and Nudt21-cKO mice analyzed at 6 hours post-LPS injection (n=5 per group).

**(H-I)** Representative flow cytometry plots (H) and frequency of dying cells (Annexin V+7-AAD+) among splenic macrophages (I) from WT and Nudt21-cKO mice analyzed 6 hours after LPS injection (n=10 per group).

**(J)** Quantification of the percentage of 7-AAD+ WT and Nudt21-cKO BMDMs primed with 50 ng/ml IFNγ overnight and stimulated with 50 ng/ml LPS for 24 hours (n=3 per group).

The error bars represent the standard errors of the means (SEMs). P values were determined by unpaired Student’s t test.

## Fig. S4: Decreased proinflammatory cytokine production in the macrophages of Nudt21-cKO mice in both the HLH and colitis models

**(A-B)** Histograms showing the IL12β gMFI in (A) bone marrow monocytes (BMMs) from WT and Nudt21-cKO mice analyzed at 6 hours post-LPS injection (n=4 per group). (B) Peritoneal monocyte-derived macrophages from WT and Nudt21-cKO mice were analyzed 6 hours after LPS injection (n=5 per group).

**(C-D)** Frequency of TNFα-producing cells (C) and quantification of the TNFα gMFI (D) in the intestinal macrophages of WT and Nudt21-cKO mice on day 8 after DSS treatment (n=4 per group).

**(E-F)** Frequency of IL6-producing cells (E) and quantification of the IL6 gMFI (F) in the intestinal macrophages of WT and Nudt21-cKO mice on day 8 after DSS treatment (n=5 per group).

The error bars represent the standard errors of the means (SEMs). P values were determined by unpaired Student’s t test.

## Fig. S5: Enhanced Autophagy Activity in Nudt21-Ablated Macrophages

**(A)** Histogram summarizing changes in the mRNA expression levels of WT and Nudt21-cKO BMDMs primed with 50 ng/ml IFNγ overnight and stimulated with 50 ng/ml LPS for the indicated durations.

**(B)** MA plot showing differential gene expression in WT and Nudt21-cKO BMDMs primed with 50 ng/ml IFNγ overnight and stimulated with 50 ng/ml LPS for 4 hours. The upregulated genes in Nudt21-cKO are marked in red, the downregulated genes in blue, and the nonsignificant genes in gray.

**(C)** Gene Ontology (GO) enrichment analysis of genes upregulated in Nudt21-cKO BMDMs compared with WT control cells upon IFNγ/LPS activation at 4 hours.

**(D)** KEGG pathway analysis showing enrichment of the autophagy pathway in genes upregulated in Nudt21-cKO BMDMs compared with WT BMDMs upon IFNγ/LPS activation at 4 hours.

**(E)** Statistical analysis of the geometric mean fluorescence intensity (gMFI) in monodansylcadaverine (MDC) assays of WT and Nudt21-cKO BMDMs primed with 50 ng/ml IFNγ overnight and stimulated with 50 ng/ml LPS for the indicated durations.

**(F)** GO enrichment analysis of genes with shortened 3’UTRs in Nudt21-cKO BMDMs compared with WT BMDMs primed with 50 ng/ml IFNγ overnight and stimulated with 50 ng/ml LPS for 4 hours.

**(G)** GO enrichment analysis of 354 genes with changes in the percent distal polyA site usage index (PDUI) and mRNA levels in Nudt21-cKO BMDMs compared with WT control cells.

**(H)** RT‒qPCR analysis of Wipi2 mRNA decay in WT and Nudt21-cKO BMDMs at 1 hour and 2 hours after 5 μM Actinomycin D (ActD) treatment (n=3 per group).

**(I)** Fluorescence intensity ratios of JC-1-Red and JC-1-Green in WT and Nudt21-cKO BMDMs upon IFNγ/LPS activation with and without BafA1. (8 h, n=3 per group).

The error bars represent the standard errors of the means (SEMs). P values were determined by unpaired Student’s t test.

## Fig. S6: Schematic diagram of the biological role of alternative mRNA polyadenylation in regulating macrophage hyperactivation via the autophagy pathway.

This schematic diagram depicts the proposed biological function of Nudt21-mediated alternative mRNA polyadenylation in regulating the stability of key autophagy gene mRNAs through 3’UTR polyA site selection. This mechanism further influences the anti-inflammatory role of autophagy in macrophage-induced hyperinflammatory diseases.

# Supplemental Material

Table S1: Antibodies used for ﬂow cytometry.

| **Antibody** | **Clone** | **Cat No.** | **Brand** | **Dilution** |
| --- | --- | --- | --- | --- |
| TNFa FITC | MP6-XT22 | 506304 | Biolegend | 1:150 |
| TNFa BV786 | MP6-XT22 | 506341 | Biolegend | 1:150 |
| TNFa PE | MP6-XT22 | 506306 | Biolegend | 1:150 |
| IL-12/23 p40 PE | C17.8 | 12-7123-82 | eBioscience | 1:180 |
| IL6 PE | MP5-20F3 | 504504 | Biolegend | 1:150 |
| CD45.2 APC-CY7 | 104 | 109824 | Biolegend | 1:300 |
| CD45.2 APC/Fire™ 750 | 30-F11 | 103154 | Biolegend | 1:300 |
| CD11b BV650 | M1/70 | 101239 | Biolegend | 1:300 |
| I-A/I-E AF700 | M5/114.15.2 | 107622 | Biolegend | 1:300 |
| CD4 PB | GK1.5 | 100428 | Biolegend | 1:300 |
| CD8 PerCP-CY5.5 | 53-6.7 | 100734 | Biolegend | 1:300 |
| CD45R/B220 PE | RA3-6B2 | 103208 | Biolegend | 1:300 |
| TCRb PE-Cy7 | H57-597 | 109222 | Biolegend | 1:300 |
| NK1.1 APC | PK136 | 108710 | Biolegend | 1:300 |
| CD11C BV421 | N418 | 117330 | Biolegend | 1:300 |
| CD11C APC | N418 | 117310 | Biolegend | 1:300 |
| Ly6C BV785 | HK1.4 | 128041 | Biolegend | 1:300 |
| Ly6C FITC | HK1.4 | 128006 | Biolegend | 1:300 |
| Ly6G BV711 | 1A8 | 127643 | Biolegend | 1:300 |
| Ly6G PE | 1A8 | 551461 | BD Pharmingen | 1:300 |
| F4/80 FITC | BM8 | 123108 | Biolegend | 1:300 |
| F4/80 APC | BM8 | 123116 | Biolegend | 1:300 |
| F4/80 PE-CY7 | BM8 | 123114 | Biolegend | 1:300 |
| CD3 PE-CY7 | 17A2 | 100220 | Biolegend | 1:300 |
| CD3 BV785 | 145-2C11 | 100355 | Biolegend | 1:200 |
| CD3 PB | 17A2 | 100214 | Biolegend | 1:300 |
| CD19 PB | 6D5 | 115523 | Biolegend | 1:300 |
| CD19 PE-CY7 | 6D5 | 115520 | Biolegend | 1:300 |

Table S2: Primers used for qRT‒PCR.

| **Target** | **Sequence (5'-3')** |
| --- | --- |
| *Nudt21* Forward | GATTGGGATGAGAAGGACTGTAG |
| *Nudt21* Reverse | TTCAACTTCATCTTCTCCTGGG |
| *Cpsf6 Forward* | GCCAACATCAGATAGTCGAGGTC |
| *Cpsf6 Reverse* | GCTTCACTCAGAGGTGTTCTTGC |
| *Cpsf7 Forward* | CCTCCACCTTCTACAGTGAGTG |
| *Cpsf7 Reverse* | GTGAGTAGTGTCTCAATGGCGTC |
| *Gabarap Forward* | CAAAGAGGAGCATCCGTTCGAG |
| *Gabarap Reverse* | TTGTCCAGGTCTCCTATCCGAG |
| *Gabarapl1 Forward* | GTGGAGAAGGCTCCTAAAGCCA |
| *Gabarapl1 Reverse* | AGGTCTCAGGTGGATCCTCTTC |
| *Atg7 Forward* | CCTGTGAGCTTGGATCAAAGGC |
| *Atg7 Reverse* | GAGCAAGGAGACCAGAACAGTG |
| *Atg12 Forward* | GAAGGCTGTAGGAGACACTCCT |
| *Atg12 Reverse* | GGAAGGGGCAAAGGACTGATTC |
| *Wipi2 Forward* | AGGACGCCAACTTAGAAGCC |
| *Wipi2 Reverse* | CATGGGAGGATGTTCGCTGT |
| *Ulk2 Forward* | CAGCACTCTGCTGTAGCACT |
| *Ulk2 Reverse* | CTGGGATGGGTGACAGAACC |
| *Sqstm1 Forward* | GCTCTTCGGAAGTCAGCAAACC |
| *Sqstm1 Reverse* | GCAGTTTCCCGACTCCATCTGT |
| *18 s Forward* | TAGAGGGACAAGTGGCGTTC |
| *18 s Reverse* | CGCTGAGCCAGTCAGTGT |
| *Tubb5 Forward* | TATGGACTCCGTTCGCTCAGGT |
| *Tubb5 Reverse* | CCTTTAGCCCAGTTGTTGCCTG |
| *Actb Forward* | CTTCTTTGCAGCTCCTTCGTT |
| *Actb Reverse* | AGGAGTCCTTCTGACCCATTC |
| *Il6 Forward* | CCACTTCACAAGTCGGAGGC |
| *Il6 Reverse* | TTCTGCAAGTGCATCATCGT |
| *Tnfa Forward* | TCTTCTCATTCCTGCTTGTGG |

| *Tnfa Reverse* | GGTCTGGGCCATAGAACTGA |
| --- | --- |
| *Il1b Forward* | TGCCACCTTTTGACAGTGATG |
| *Il1b Reverse* | TGATGTGCTGCTGCGAGATT |
| *Map1lc3b Forward* | GTCCTGGACAAGACCAAGTTCC |
| *Map1lc3b Reverse* | CCATTCACCAGGAGGAAGAAGG |
| *Map1lc3b (distal PAS) Forward* | CAGAAGAGCAGTGTCAGGGG |
| *Map1lc3b (distal PAS) Reverse* | GCTGAGTGGGAGCCCTTTTA |
| *Ulk2 (distal PAS) Forward* | AGCCAGATGCTTCCTTAGAGA |
| *Ulk2 (distal PAS) Reverse* | GTTTTGAATCCTGGAGCAGGG |
